# Liver Steatosis is Prevalent in Lean People With HIV and Associated With Exposure to Antiretroviral Treatment—A Cross-sectional Study

**DOI:** 10.1093/ofid/ofae266

**Published:** 2024-05-07

**Authors:** Louise E van Eekeren, Nadira Vadaq, Wilhelm A J W Vos, Marc J T Blaauw, Albert L Groenendijk, Jan van Lunzen, Janneke E Stalenhoef, Marvin A H Berrevoets, Annelies Verbon, Gert Weijers, Mihai G Netea, André J A M van der Ven, Quirijn de Mast, Leo A B Joosten, Eric T T L Tjwa

**Affiliations:** Department of Internal Medicine and Radboud Center for Infectious Diseases, Radboud University Medical Center, Nijmegen, The Netherlands; Department of Internal Medicine and Radboud Center for Infectious Diseases, Radboud University Medical Center, Nijmegen, The Netherlands; Department of Internal Medicine, OLVG, Amsterdam, The Netherlands; Department of Internal Medicine and Radboud Center for Infectious Diseases, Radboud University Medical Center, Nijmegen, The Netherlands; Department of Internal Medicine, Elisabeth-Tweesteden Hospital, Tilburg, The Netherlands; Department of Medical Microbiology and Infectious Diseases, Erasmus MC, The Netherlands; Department of Internal Medicine and Radboud Center for Infectious Diseases, Radboud University Medical Center, Nijmegen, The Netherlands; Department of Internal Medicine, OLVG, Amsterdam, The Netherlands; Department of Internal Medicine, Elisabeth-Tweesteden Hospital, Tilburg, The Netherlands; Department of Medical Microbiology and Infectious Diseases, Erasmus MC, The Netherlands; Medical UltraSound Imaging Center (MUSIC), Division of Medical Imaging, Radboud University Medical Center, Nijmegen, The Netherlands; Department of Internal Medicine and Radboud Center for Infectious Diseases, Radboud University Medical Center, Nijmegen, The Netherlands; Department of Metabolism and Immunology, Life and Medical Sciences Institute, University of Bonn, Bonn, Germany; Department of Internal Medicine and Radboud Center for Infectious Diseases, Radboud University Medical Center, Nijmegen, The Netherlands; Department of Internal Medicine and Radboud Center for Infectious Diseases, Radboud University Medical Center, Nijmegen, The Netherlands; Department of Internal Medicine and Radboud Center for Infectious Diseases, Radboud University Medical Center, Nijmegen, The Netherlands; Department of Medical Genetics, Iuliu Hatieganu University of Medicine and Pharmacy, Cluj-Napoca, Romania; Department of Gastroenterology and Hepatology, Radboud University Medical Centre, Nijmegen, The Netherlands

**Keywords:** HIV, Lean, MASLD, Steatosis

## Abstract

**Background:**

Steatotic liver disease is suggested to have a higher prevalence and severity in people with HIV (PHIV), including in those with a normal body mass index (BMI). In this study, we used data from the 2000HIV cohort to (1) assess the prevalence of liver steatosis and fibrosis in lean versus overweight/obese PHIV and (2) assess associations in these subgroups between steatosis and fibrosis with traditional risk factors and HIV-specific characteristics.

**Methods:**

The 2000HIV study cohort comprises 1895 virally suppressed PHIV that were included between 2019 and 2021 in 4 HIV treatment centers in the Netherlands. The majority (58.5%) underwent vibration-controlled transient elastography for the assessment of liver steatosis and fibrosis. The prevalence of steatosis (controlled attenuation parameter ≥263 dB/m) and fibrosis (liver stiffness measurement ≥7.0 kPa) was estimated. Multiple factors including HIV characteristics and antiretroviral drugs were tested in a logistic regression model for association with steatosis and fibrosis. Analyses were performed separately for lean (Asian descent: BMI < 23 kg/m^2^, other descent: BMI < 25 kg/m^2^) and overweight/obese (other BMI) participants.

**Results:**

Of 1050 PHIV including 505 lean and 545 overweight/obese PHIV, liver steatosis was observed in 37.7% of the overall study population, 19.7% of lean, and 54% of overweight/obese PHIV, whereas fibrosis was observed in 9.0% of the overall study population, 5.9% of lean, and 12.0% of overweight/obese PHIV.

All associations with fibrosis and most associations with steatosis concerned metabolic factors such as type 2 diabetes mellitus (overall population: adjusted odds ratio [aOR] for steatosis: 2.3 [1.21-4.4], *P* = .011; aOR for fibrosis: 3.7 [1.82-7.53], *P* < .001). Furthermore, in lean PLHIV, liver steatosis was associated with CD4 and CD8 counts at enrollment, dual therapy, and history of treatment with raltegravir (aOR: 3.6 [1.53-8.47], *P* = .003), stavudine (aOR: 3.73 [1.69-8.2], *P* = .001), and indinavir (aOR: 3.86 [1.59-9.37], *P* = .003). These associations were not observed in overweight/obese PHIV.

**Conclusions:**

Liver steatosis was highly prevalent, affecting approximately one-fifth of lean PHIV and half of overweight/obese PHIV. Fibrosis was observed in a minority. Both steatosis and fibrosis were associated with traditional metabolic risk factors. In addition, (prior) exposure to specific antiretroviral drugs was associated liver steatosis in lean, but not in overweight/obese PHIV. Implementing increased screening protocols could enhance the identification of steatotic liver disease in lean PHIV.

## Teaser

- One-fifth of lean PHIV has liver steatosis
- Metabolic factors were associated with steatosis in both lean and overweight/obese PHIV
- Exposure to specific antiretroviral drugs is associated with liver steatosis in lean, but not in overweight/obese PHIV

Although the introduction of combination antiretroviral therapy (cART) has increased the life expectancy of people with HIV (PHIV), liver disease has emerged as 1 of the leading causes of death in the aging population of PHIV [1]. Metabolic dysfunction-associated steatotic liver disease (MASLD), formerly known as nonalcoholic fatty liver disease (NAFLD), is a major health concern [2, 3], being now the most common cause of liver disease in PHIV [4]. The global prevalence of NAFLD in the general population is estimated to be 25% [5], whereas among PHIV estimates range from 13% to 70% [6, 7]. The prevalence of MASLD in the general population is thought to be similar to the prevalence of NAFLD [3]. MASLD is characterized by steatosis, which is the accumulation of fat in hepatic tissue of more than 5% of the liver weight. Simple steatosis can progress to steatohepatitis, fibrosis, cirrhosis, and patients, especially those with a lean body, are at an increased risk of cardiovascular diseases [8–10].

Obesity and related metabolic disorders are closely associated with liver steatosis. Like the general population, metabolic disorders such as obesity, insulin resistance, hypertension, and dyslipidemia have been identified as risk factors for liver steatosis in PHIV [7]. However, PHIV may develop liver steatosis at lower body mass indexes (BMI) compared to the general population [11]. Liver steatosis in lean individuals (ie, those are not overweight or obese) may be a prevalent condition among PHIV [12]. Because lean individuals lack the classical risk factor of an elevated BMI, the factors driving liver steatosis in lean PHIV may differ from those in overweight/obese PHIV.

In addition to traditional metabolic risk factors, HIV-specific characteristics and exposure to antiretroviral drugs may contribute to the development and progression of liver steatosis in PHIV. HIV is hypothesized to contribute to liver steatosis through metabolic alterations [13] and fibrogenesis by inducing changes in hepatic cells, including Kupffer cells and hepatic stellate cells [14]. However, associations between both steatosis [7, 15] and fibrosis [15, 16] with HIV-specific characteristics such as CD4+ T-cell counts, HIV duration, and detectable HIV-RNA viral load during cART have been inconsistent. Exposure to specific antiretroviral drugs may also play a role in steatogenesis and fibrogenesis in PHIV. For example, exposure to dideoxynucleoside analogs (d-drugs) may predispose individuals to liver steatosis and fibrosis through their effects on mitochondrial dysfunction [6, 16], whereas exposure to integrase strand transfer inhibitors (INSTIs) and tenofovir-alafenamide may contribute to liver steatosis through weight gain effects [17].

Particularly in lean individuals, the drivers of liver steatosis remain unclear. Therefore, our study aimed to assess risk factors for liver steatosis in a large cohort of PHIV, including subgroups of lean and overweight/obese PHIV. In the 2000HIV study, a Dutch real-world study comprising nearly 2000 virally suppressed PHIV, participants underwent vibration-controlled transient elastography (VCTE) with controlled attenuation parameter (CAP) to assess steatosis and fibrosis. Our specific objectives were 2-fold: (1) to determine the prevalence of liver steatosis and fibrosis in both lean and overweight/obese PHIV and (2) to examine the associations between various demographic-, metabolic-, environmental-, and HIV-specific factors, including current and previous antiretroviral drugs regimens, and steatosis and fibrosis, in the entire study population as well as in the subgroups of lean and overweight/obese PHIV.

## METHODS

### Study Design

The study design of the 2000HIV cohort has been previously described elsewhere [18]. In short, we conducted a longitudinal observational study in 4 HIV treatment centers in the Netherlands: Radboud University Medical Center Nijmegen, Erasmus Medical Center Rotterdam, OLVG Amsterdam, and Elisabeth-Twee-Steden Hospital Tilburg. Participants with HIV were included in the study between October 2019 and October 2021. Inclusion criteria were HIV-1 infection, age ≥ 18 years, cART duration ≥ 6 months, and HIV-RNA load <200 copies/mL. Elite controllers (as previously defined [18]) were also eligible for inclusion. Exclusion criteria were detectable viral hepatitis B or C DNA by polymerase chain reaction, signs or symptoms of other active infections, and pregnancy.

### Patient Consent Statement

The 2000HIV study protocol was approved by the Independent Review Board Nijmegen (ref. NL68056.091.81) and published at clinicaltrials.gov (ID: NCT03994835). The study was conducted in accordance with the principles of the Declaration of Helsinki. All study participants provided written informed consent before inclusion.

### Data Extraction and Questionnaires

For the current analysis, we used a cross-sectional design because we only used data from the baseline visit of the 2000HIV cohort. Demographic data, clinical data including history of HIV and comorbidities, current cART regimens, comedication, and biometric data were registered. Details can be found in the Supplementary files.

#### Definitions

BMI was categorized according to the National Institutes of Health criteria [19]: underweight: BMI < 18.5 kg/m^2^; normal weight: BMI ≥18.5 and <25 kg/m^2^; overweight: BMI ≥25 and <30 kg/m^2^; and obese: BMI ≥30 kg/m^2^. In people of Asian descent, BMI was classified as [20]: underweight: BMI < 18.5 kg/m^2^; normal weight: BMI ≥18.5 and <23 kg/m^2^; overweight: BMI ≥ 23 and <25 kg/m^2^; and obese: BMI ≥ 25 kg/m^2^. The subgroup of lean participants consisted of PHIV that were underweight or of normal weight, whereas the overweight/obese subgroup consisted of PHIV that were overweight or obese.

Metabolic syndrome was defined using a modified National Cholesterol Education Program definition [21] as the presence of ≥3 of the following 5 traits: (1) abdominal obesity, defined as a BMI ≥ 30 kg/m^2^ (corresponding to a waist circumference ≥102 cm [40 in] in men and ≥88 cm [35 in] in females) [22]; (2) serum triglycerides ≥1.7 mmol/L or lipid-lowering therapy (LLT); (3) serum high-density lipoprotein (HDL) cholesterol <1 mmol/L in males and <1.3 mmol/L in females or LLT; (4) systolic blood pressure ≥130, diastolic blood pressure 85 ≥mm Hg or antihypertensive medication; and (5) previously diagnosed type 2 diabetes.

### Vibration-controlled Transient Elastography

The liver stiffness measurement (LSM) and CAP were measured in >4-hour fasting participants by trained operators using VCTE with an M and/or XL probe (FibroScan, Echosens, Paris, France). Participants were placed in a supine position with the right arm in abduction. Measurements were taken in an intercostal space at the intersection of the right midaxillary line and a transverse line at the level of the xiphoid process. The measurements were considered reliable if at least 10 valid measurements were obtained, with an interquartile range of <30% from the median measurement. The results were expressed as kPa for LSM and dB/m for CAP. Cutoff values for steatosis and fibrosis grades were based on studies performed in patients with NAFLD. S0 (no steatosis) was defined as CAP < 263 dB/m, S1 (mild steatosis) was defined as CAP ≥ 263 dB/m and <280 dB/m, and S2/S3 (moderate to severe steatosis) was defined as CAP ≥ 280 dB/m [23, 24]. F0-F1 (no-mild fibrosis) was defined as LSM < 7.0 kPa, F2 (moderate fibrosis) was defined as LSM ≥ 7.0 and <8.7 kPa, F3 (severe fibrosis) was defined as LSM ≥ 8.7 and <10.3 kPa, and F4 (cirrhosis) was defined was LSM ≥ 10.3 kPa [25].

Combining BMI categories and CAP cutoffs, we distinguish the following groups: lean (under- or normal weight BMI) with steatosis (CAP ≥ 263 dB/m); lean without steatosis; overweight/obese (overweight or obese BMI) with steatosis; and overweight/obese without steatosis. Similarly, we distinguish lean and overweight/obese PHIV with and without fibrosis: lean with fibrosis (LSM ≥ 7.0); lean without fibrosis; overweight/obese with fibrosis; and overweight/obese without fibrosis.

### Statistical Analysis

Continuous variables are summarized as median and interquartile range and categorical variables are shown as number and percentage. Associations between continuous variables are assessed by Spearman's correlation analysis for nonnormally distributed data. Associations between categorical variables (ie, physical activity) and the presence of steatosis and/or fibrosis was tested using chi-squared tests. Strength of correlations between 2 binary variables were calculated using Phi correlation coefficients. Initially, we assessed associations between demographic factors and steatosis (CAP ≥ 248 dB/m) and fibrosis (LSM ≥ 7.0) using univariate logistic regression. Next, we performed multivariate logistic regression for comorbidities, laboratory measurements, HIV characteristics, and antiretroviral treatment (ART) exposure. Demographic characteristics that were significantly associated with steatosis or fibrosis in a univariate logistic regression model were added as confounders in these multivariate models. In addition, LLT was added as a confounder to the model for the laboratory measurements. The specific variables tested are presented in Supplementary Table 1. Finally, we aimed to assess which factors remained predictive of steatosis and fibrosis in a multivariate model. Variables that were significantly associated with steatosis or fibrosis from the model corrected for demographic characteristics were selected for the final multivariate logistic regression models to assess predictors of steatosis and fibrosis, respectively. To avoid multicollinearity, all variables with a variance inflation factor >10 except 1 were removed from the models. *P* values below .05 were considered significant. Significance levels are depicted with asterisks: *P .05–.01; **P value .01–.001; and ***P < .001. Statistical analysis was performed using the R software version 4.1.3 (R core team, Vienna, Austria).

## RESULTS

### Study Population

**Figure 1.**
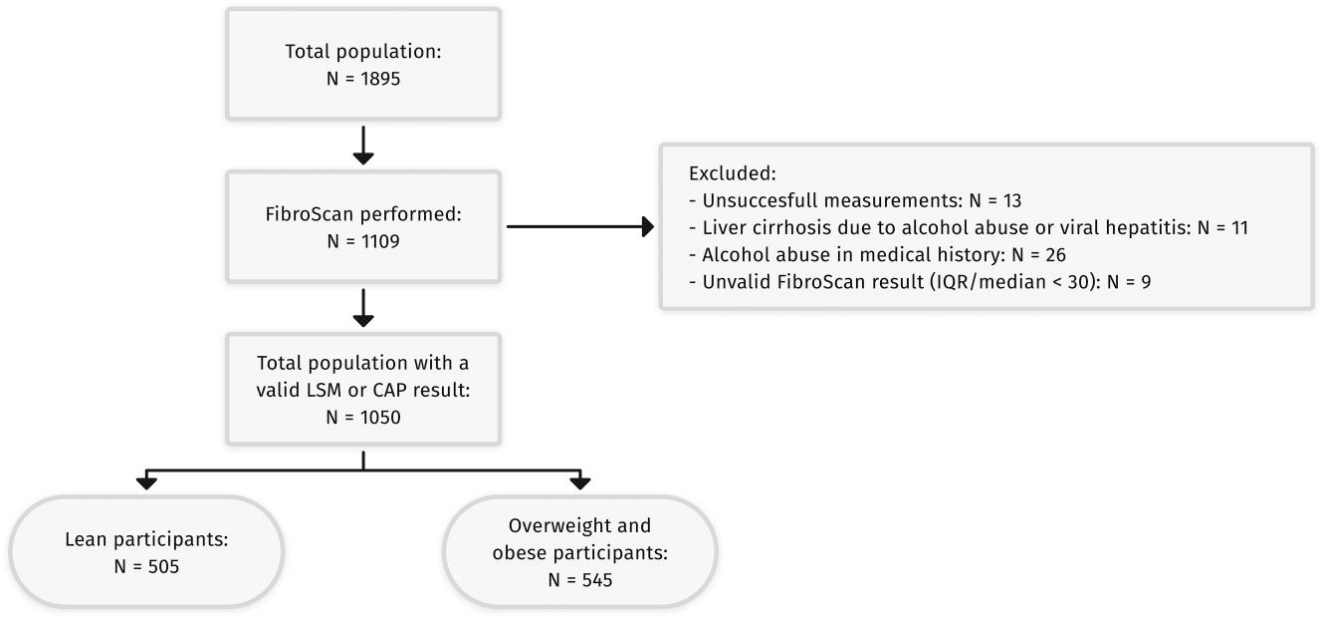
Flow diagram of the study population. Abbreviations: CAP, controlled attenuation parameter; LSM, liver stiffness measurement.

Between 2019 and 2021, 1895 participants were recruited. VCTE using FibroScan was performed in a subset (n = 1109) depending on availability of the FibroScan at the inclusion sites (Figure 1). After excluding participants with unsuccessful measurements, cirrhosis resulting from alcohol abuse or viral hepatitis, or a history of alcohol abuse, 1050 including 505 “lean” and 545 “overweight/obese” PHIV remained. Baseline characteristics are shown in Table 1 and Supplementary Table 2 (ART history). The lean group consisted of fewer females, more participants of European ancestry, more current smokers, and were slightly younger compared to the overweight/obese group. As expected, they differed in CAP and LSM and in metabolic characteristics. Regarding HIV- and ART-specific characteristics, the groups differed in transmission route, CD8 count at enrollment (lower in lean PHIV), and ART history (more exposure to d-drugs and indinavir [IDV] in lean PHIV) as well as current cART regimen (less dual therapy in lean PHIV) (Table 1).

**Table 1.** Baseline Characteristics

Variable	Total Population, N = 1050	BMI Classification		
		Lean, N = 505	Overweight or Obese, N = 545	
Female sex	143 (14%)	47 (9.3%)	96 (18%)	**<.001**
Age	52 (43–59)	51 (41–59)	52 (45–60)	**.008**
Ethnicity				
Asian	40 (3.8%)	17 (3.4%)	23 (4.2%)	
Black	107 (10%)	36 (7.2%)	71 (13%)	
Hispanic	24 (2.3%)	13 (2.6%)	11 (2.0%)	
Mixed	57 (5.4%)	18 (3.6%)	39 (7.2%)	
Native American	3 (0.3%)	2 (0.4%)	1 (0.2%)	
White	817 (78%)	417 (83%)	400 (73%)	
BMI (kg/m^2^)	25.1 (22.7–27.8)	22.6 (21.2–23.9)	27.7 (26.1–30.2)	**<.001**
Smoker (current)	310 (30%)	174 (34%)	136 (25%)	**<.001**
Pack-years	21 (10-35)	21 (8-33)	22 (12-37)	.31
CAP (dB/m)	245 (212–286)	224 (198–255)	270 (235–304)	**<.001**
LSM (kPa)	4.40 (3.70–5.50)	4.20 (3.50–5.10)	4.70 (3.90–5.90)	**<.001**
Hypertension	262 (25%)	94 (19%)	168 (31%)	**<.001**
T2DM	52 (5.0%)	18 (3.6%)	34 (6.2%)	**.046**
Lipid-lowering therapy	215 (20%)	87 (17%)	128 (23%)	**.012**
Metabolic syndrome	271 (26%)	81 (16%)	190 (35%)	**<.001**
Prior infection with HBV	317 (31%)	142 (29%)	175 (33%)	.14
Prior infection with HCV	79 (7.5%)	39 (7.7%)	40 (7.3%)	.81
Total cholesterol (mmol/L)	5.11 (4.35–5.76)	5.13 (4.32–5.67)	5.09 (4.36–5.81)	.74
HDL (mmol/L)	1.16 (0.98–1.43)	1.24 (1.01–1.48)	1.12 (0.96–1.34)	**<.001**
LDL (mmol/L)	2.10 (1.78–2.45)	2.08 (1.73–2.42)	2.11 (1.80–2.46)	.13
VLDL (mmol/L)	0.79 (0.60–1.00)	0.74 (0.58–0.94)	0.83 (0.63–1.04)	**<.001**
Triglycerides (mmol/L)	1.18 (0.86–1.71)	1.09 (0.82–1.45)	1.35 (0.90–1.90)	**<.001**
ALT (U/L)	25 (19–34)	23 (18–30)	27 (21–36)	**<.001**
AST (U/L)	26 (22–31)	25 (20–31)	27 (22–32)	.13
ALP (U/L)	79 (66–94)	80 (65–95)	79 (66–94)	.90
GGT (U/L)	28 (19–49)	24 (17–43)	32 (21–54)	**.003**
Glucose (mmol/L)	5.50 (5.10–6.00)	5.50 (5.00–5.80)	5.60 (5.10–6.20)	**.018**
HIV duration (y)	11 (6–17)	11 (6–16)	11 (6–17)	.65
Transmission route				**<.001**
Blood products	4 (0.4%)	1 (0.2%)	3 (0.6%)	
Congenital	8 (0.8%)	5 (1.0%)	3 (0.6%)	
Heterosexual	238 (23%)	85 (17%)	153 (29%)	
IV drug use	8 (0.8%)	3 (0.6%)	5 (0.9%)	
MSM	743 (72%)	389 (78%)	354 (66%)	
Unknown	33 (3.2%)	18 (3.6%)	15 (2.8%)	
Nadir CD4 count (10^6^ cells/L)	0.27 (0.15–0.41)	0.27 (0.16–0.41)	0.26 (0.13–0.39)	.25
CD4:CD8 ratio pre-ART	0.28 (0.17–0.47)	0.30 (0.19–0.50)	0.27 (0.16–0.44)	**.018**
HIV-RNA zenith (copies/mL)	100 000 (39,991–253 848)	100 000 (41,000–247 802)	100 000 (38,270–256 933)	.96
AIDS-defining wasting in MH	101 (9.6%)	40 (7.9%)	61 (11%)	.072
CD4 at enrollment (10^6^ cells/L)	0.68 (0.50–0.87)	0.66 (0.50–0.84)	0.71 (0.51–0.90)	.16
CD8 at enrollment (10^6^ cells/L)	0.81 (0.61–1.12)	0.79 (0.59–1.08)	0.86 (0.63–1.17)	**.020**
CD4:CD8 ratio at enrollment	0.89 (0.62–1.20)	0.92 (0.66–1.27)	0.83 (0.59–1.17)	.081
ART duration (y)	9 (5–15)	9 (5–16)	9 (5–15)	.93
No ART at enrollment	15 (1.4%)	7 (1.4%)	8 (1.5%)	.91
Dual therapy at enrollment	147 (14%)	59 (12%)	88 (16%)	**.037**
NRTI in current regimen	1005 (96%)	483 (96%)	522 (96%)	.91
NtRT in current regimen	658 (63%)	324 (64%)	334 (61%)	.34
NNRTI in current regimen	373 (36%)	194 (38%)	179 (33%)	.059
PI in current regimen	94 (9.0%)	43 (8.5%)	51 (9.4%)	.63
INSTI in current regimen	613 (58%)	284 (56%)	329 (60%)	.18

In this table, the baseline characteristics, except for ART history (Supplementary Table 2), are shown for the total group of participants with a valid CAP or LSM result, as well as for the subgroups “lean” and “overweight/obese.” Dichotomous variables are shown as number (%), and numerical variables are shown as median (Q1-Q3). The column *P* value depicts the *P* values of comparisons (Chi-square test for dichotomous variables and Mann-Whitney *U* for numerical variables) between lean and overweight/obese participants. *P* values < 0.05 are shown in bold. Ethnicity was self-identified. Abbreviations: ALT, alanine transaminase; ALP, alkaline phosphatase; ART, antiretroviral therapy; AST, aspartate aminotransferase; CAP, controlled attenuation parameter; GGT, gamma-glutamyl transferase; HBV, hepatitis B virus; HCV, hepatitis C virus; HDL, high-density lipoprotein; INSTI, integrase strand transfer inhibitor; LDL, low density lipoprotein; LSM, liver stiffness measurement; MSM, men who have sex with men; NNRTI, non-nucleoside reverse transcriptase inhibitor; NRTI, nucleoside reverse transcriptase inhibitor; NtRTI, nucleotide reverse transcriptase inhibitor; T2DM, type 2 diabetes mellitus; PI, protease inhibitor; VLDL, very low-density lipoprotein.

Of the total group (n = 1050), 46 (4.4%) were fasting less than 4 hours; for 23 participants (2.2%), the duration of fasting was unknown. Of note, 26 PHIV (26/1050 = 2.5%) received treatment for hepatitis B virus (HBV) infection at the time of inclusion (with undetectable HBV-DNA polymerase chain reaction according to inclusion criteria). In addition, 10 participants (10/1050 = 0.95%) used known steatogenic medications [26]: 4 participants used systemic glucocorticoids, 1 used methotrexate, 1 used both methotrexate and systemic glucocorticoids, and 4 used valproic acid. Because of the small numbers, these participants were not analyzed separately.

### Steatosis is Prevalent but Poorly Recognized in Lean PHIV

**Figure 2.**
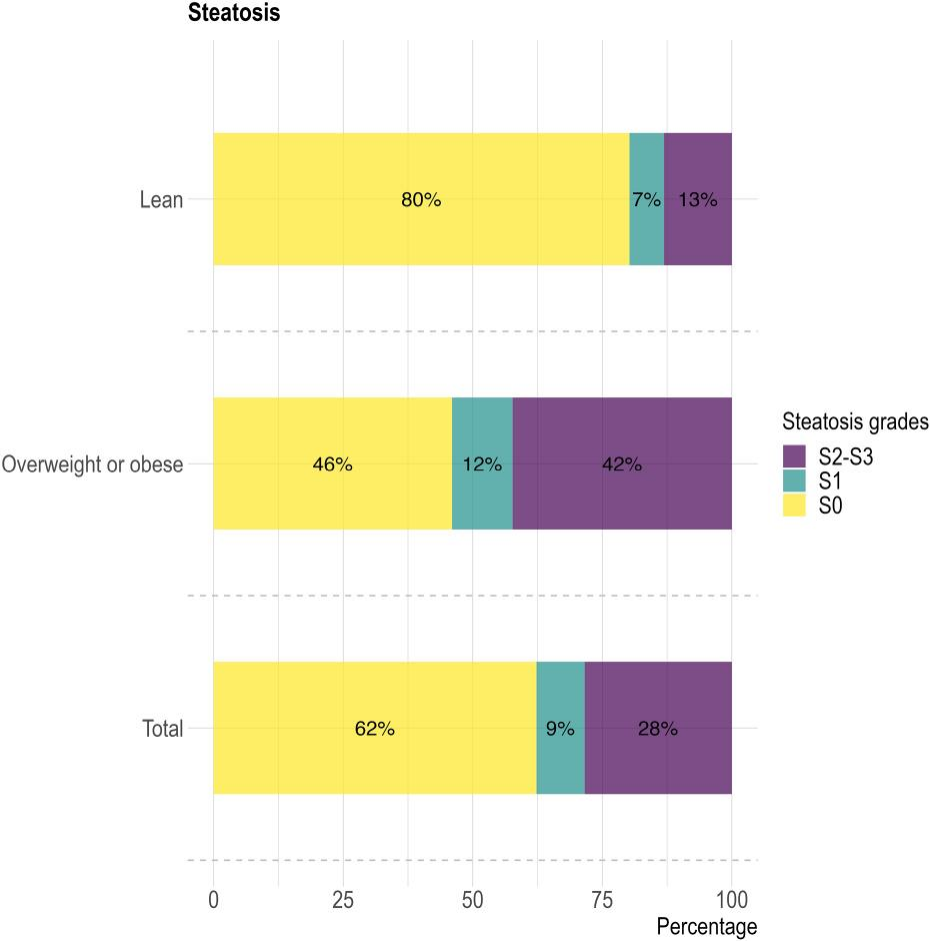
Stacked bar plots showing the prevalence of liver steatosis in the total study population (lowest bar), and the subgroups of lean (highest bar) and overweight or obese participants (middle bar). The legend shows the different steatosis grades.

Steatosis affected more than one-third of our study population: 37.7% (95% confidence interval [CI], 34.7-40.7) had significant steatosis (CAP ≥ 263). As expected, especially the group of overweight/obese participants was affected by steatosis (prevalence 54.0%), but steatosis was also common amongst lean participants with a prevalence of 19.7% (Figure 2).

Documentation of liver steatosis was limited in our population. Only 18.2% (67/369) of all participants with measured liver steatosis also had documented steatosis in their medical file. Especially in the lean group, liver steatosis was seldom recognized by the treating physician: patients’ medical history reported liver steatosis in 12.0% (11/92) of lean participants with liver steatosis versus 20.2% (56/277) in overweight or obese participants.

Liver fibrosis was less prevalent than steatosis, affecting 9.0% (95% CI, 7.3-10.8) of our total study population. Fibrosis was classified as F2 in 6.0% (95% CI, 4.5-7.5), F3 in 1.8% (95% CI, 1.0-2.7), and F4 in 1.2% (95% CI, .5-1.9). Overweight and obese participants had a fibrosis prevalence of 12.0% (95% CI, 9.2-14.9) F2 in 7.9% (95% CI, 5.5-10.2), F3 in 2.4% (95% CI, 1.0-3.7), and F4 in 1.8% (95% CI, .6-2.9. Liver fibrosis was less common in lean participants with a prevalence of 5.9% (95% CI, 3.8-8.0) (F2 in 4.0% [95% CI, 2.2-5.7], F3 in 1.3% [95% CI, .3-2.3], and F4 in 0.6% [95% CI, −.1 to 1.3]).

CAP and LSM were weakly correlated (total study population: Spearman's rho = 0.25, *P* < .001; lean: Spearman's rho = 0.12, *P* = .013; and overweight/obese: Spearman's rho = 0.27, *P* < .001). Of all participants with steatosis, only 14.4% (49/340) had fibrosis (8.0% [7/88] in lean participants and 16.7% [42/252] in overweight or obese participants). In contrast, steatosis was common among participants with fibrosis (60.5% [49/81] in total population, 30.4% [7/23] in lean participants, and 72.4% [42/58] in overweight or obese participants). When considering the total study population, steatosis and fibrosis cooccurred in 5.4% (49/913) (1.6% [7/438] of lean participants and 8.8% [42/475] of overweight or obese participants).

### Current and Lifetime Alcohol Consumption Is not Different in PLHIV With Steatosis or Fibrosis Compared to Unaffected PLHIV

Information on self-reported current and lifetime alcohol consumption was available from 903 and 901 participants, respectively. The median weekly alcohol consumption was 3.4 units. On a lifetime basis, 136 PHIV indicated that they had never consumed alcohol, 645 PHIV indicated moderate alcohol consumption, and 120 PHIV indicated periods of excessive alcohol consumption (as defined by more than 28 [men] or 21 [women] alcoholic beverages per week). Current and lifetime consumption of alcohol did not differ in PHIV with steatosis or fibrosis compared to those without in the total study population (Supplementary Figures 1 and 2**)**, nor in the subgroups of lean (steatosis, current: *P* = .47 and lifetime: *P* = .60; fibrosis, current: *P* = .43 and lifetime: *P* = .29), and overweight/obese PHIV (steatosis, current: *P* = .23 and lifetime: *P* = .52; fibrosis, current: *P* = .94 and lifetime: *P* = .71).

### Traditional Risk Factors and Exposure to Specific Antiretrovirals in Lean People, Predict Liver Steatosis in PWHIV

#### Associations Between Demographics and Liver Steatosis and Fibrosis

**Figure 3.**
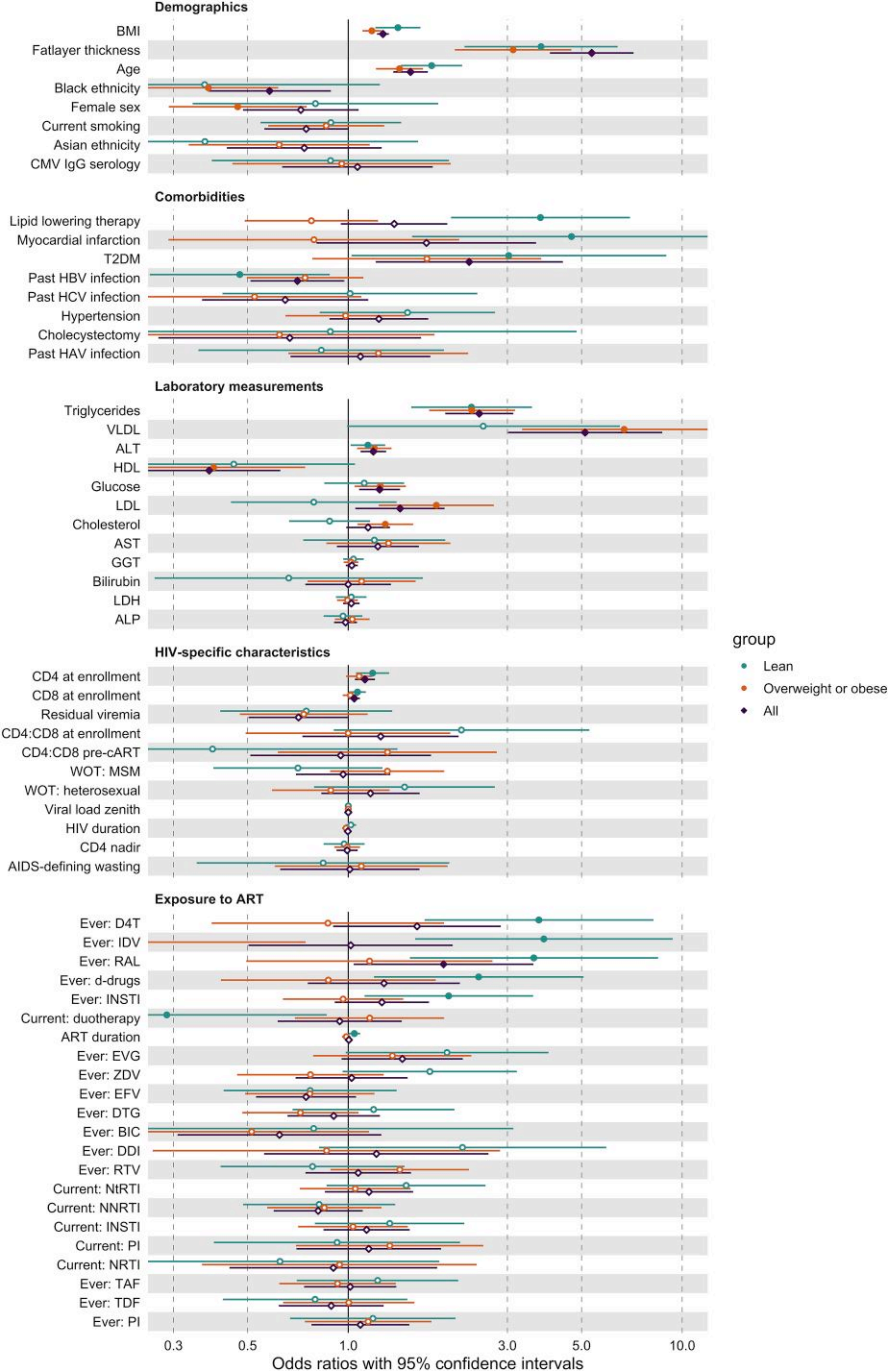
Associations between steatosis and demographics, comorbidities, laboratory measurements, HIV-specific characteristics, and ART exposure for the total population (purple), lean participants (green), and overweight and obese participants (orange). Effect estimates are presented as odds ratios with 95% confidence intervals. The odds ratios for demographics are not corrected for any confounders. The logistic regression with demographics identified 2 confounding variables: age, and fat layer thickness. Because of a strong association between BMI and fat layer thickness, only one of them was selected as a confounder for subsequent models. These confounding variables were added to the logistic regression models with comorbidities, HIV-specific characteristics, and ART exposure. In the models involving laboratory assessments, lipid-lowering therapy was also included as a confounder. Closed circles denote significant *P* values (<.05). For readability, few variables that did not show significant associations are not shown in this figure (ie, Native American ethnicity, Hispanic ethnicity, way of transmission: intravenous drugs, way of transmission: blood products, way of transmission: congenital, ever: zalcitabine, duration of untreated infection, current: no ART. Abbreviations: ALP, alkaline phosphatase; ALT, alanine transaminase; ART, antiretroviral therapy; AST, aspartate aminotransferase; BIC, bictegravir; CAP, controlled attenuation parameter; CMV, cytomegalovirus; D4T, stavudine; d-drugs, dideoxynucleoside analogs; DDI, didanosine; DTG, dolutegravir; EFV, efavirenz; EVG, elvitegravir; GGT, gamma-glutamyl transferase; HAV, hepatitis A virus; HBV, hepatitis B virus; HCV, hepatitis C virus; HDL, high-density lipoprotein; IDV, indinavir; INSTI, integrase strand transfer inhibitor; LDH, lactate dehydrogenase; LDL, low-density lipoprotein; LSM, liver stiffness measurement; MSM, men who have sex with men; NNRTI, nonnucleoside reverse transcriptase inhibitor; NRTI, nucleoside reverse transcriptase inhibitor; NtRTI, nucleotide reverse transcriptase inhibitor; RAL, raltegravir; PI, protease inhibitors; RTV, ritonavir; T2DM, type 2 diabetes mellitus; TAF, tenofovir alafenamide; TDF, tenofovir disoproxil fumarate; VLDL, very low-density lipoprotein; WOT, way of transmission; ZDV, zidovudine.

**Figure 4.**
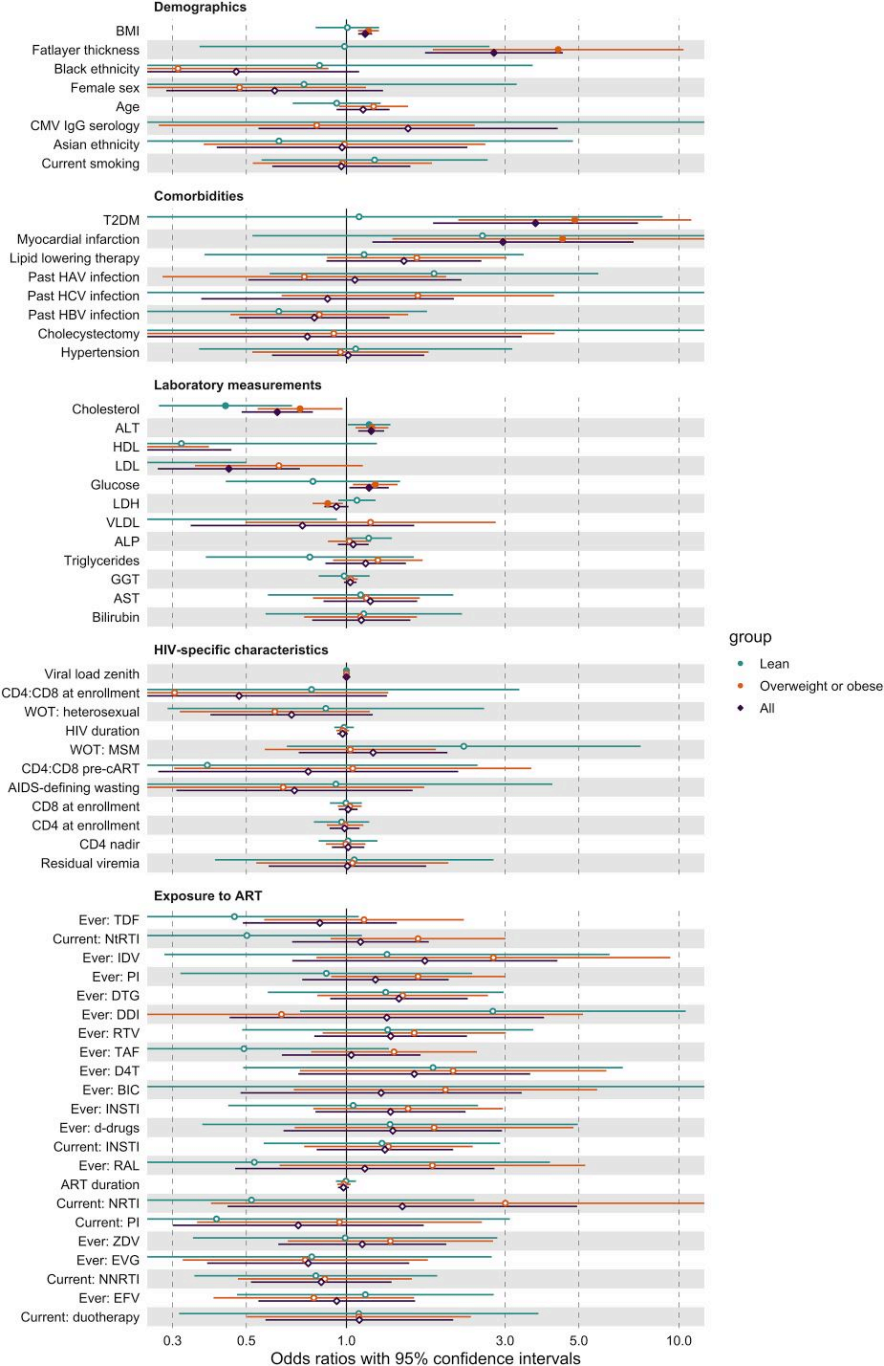
Associations between fibrosis and demographics, comorbidities, laboratory measurements, HIV-specific characteristics, and ART exposure for the total population (purple), lean participants (green), and overweight and obese participants (orange). Effect estimates are presented as odds ratios with 95% confidence intervals. The odds ratios for demographics are not corrected for any confounders. The logistic regression with demographics identified 2 confounding variables: age and fat layer thickness (BMI and fat layer thickness are strongly associated so we choose 1 of them to add as confounder in subsequent models). These confounding variables were added to the logistic regression models with comorbidities, HIV-specific characteristics, and ART exposure. For the models with laboratory assessments, we also added lipid-lowering therapy as a confounder. Closed circles denote significant *P* values (<.05). For readability, few variables that did not show significant associations are not shown in this figure (ie, Native American ethnicity, Hispanic ethnicity, way of transmission: intravenous drugs, way of transmission: blood products, way of transmission: congenital, ever: zalcitabine, duration of untreated infection, current: no ART). Abbreviations: ALT, alanine transaminase; ALP, alkaline phosphatase; ART, antiretroviral therapy; AST, aspartate aminotransferase; BIC, bictegravir; BMI, body mass index; CAP, controlled attenuation parameter; CMV, cytomegalovirus; D4T, stavudine; d-drugs, dideoxynucleoside analogs; DDI, didanosine; DTG, dolutegravir; EFV, efavirenz; EVG, elvitegravir; GGT, gamma-glutamyl transferase; HAV, hepatitis A virus; HBV, hepatitis B virus; HCV, hepatitis C virus; HDL, high-density lipoprotein; IDV, indinavir; INSTI, integrase strand transfer inhibitor; LDH, lactate dehydrogenase; LDL, low-density lipoprotein; LSM, liver stiffness measurement; MSM, men who have sex with men; NNRTI, nonnucleoside reverse transcriptase inhibitor; NRTI, nucleoside reverse transcriptase inhibitor; NtRTI, nucleotide reverse transcriptase inhibitor; PI, protease inhibitors; RAL, raltegravir; RTV, ritonavir; T2DM, type 2 diabetes mellitus; TAF, tenofovir alafenamide; TDF, tenofovir disoproxil fumarate; VLDL, very low-density lipoprotein; WOT, way of transmission; ZDV, zidovudine.

Associations with demographic factors, comorbidities, laboratory measurements, HIV characteristics, and ART exposure are shown in Figure 3 and Supplementary Table 3 for steatosis and Figure 4 and Supplementary Table 5 for fibrosis. Using univariate logistic regression, we found that the demographic factors age, BMI, and fat layer thickness showed similar associations with steatosis across the different groups. Notably, when different age cutoffs were applied, the highest odds ratios were found for an age cutoff of 40 years in the lean group (4.88 [95% CI, 2.07-11.53], *P*-value = .002), and at 50 years in the overweight/obese group (2.11 (95% CI, 1.47–3.04], *P* < .001) (Supplementary Table 4). Additionally, BMI and fat layer thickness were also associated with fibrosis in overweight/obese participants. Among overweight/obese participants, females and participants with a Black ethnicity exhibited lower odds of liver steatosis.

Age and fat layer thickness were selected as confounders in the multivariate logistic regression models. In addition, LLT was added as a confounder to the model for laboratory measurements.

#### Steatosis

LLT for the treatment of dyslipidemia, past myocardial infarction, and type 2 diabetes mellitus (T2DM) were associated with higher odds, whereas prior HBV infection was associated with lower odds of liver steatosis in lean participants. Instead, none of the comorbidities was associated with liver steatosis in overweight and obese participants.

In both subgroups, as well as in the total study population, high alanine transaminase (ALT) and triglyceride concentrations were significantly associated with liver steatosis. High low-density lipoprotein (LDL) and total cholesterol and low HDL concentrations were associated with steatosis in overweight/obese participants, and similar trends were observed in lean participants. Similar trends were also found between the groups for glucose: high glucose was associated with higher odds of liver steatosis but this was only statistically significant for overweight/obese participants.

High CD4+ and CD8+ T-cell counts at study enrollment were associated with higher odds of liver steatosis in lean participants. A similar trend was seen for overweight and obese participants, albeit not significant.

ART history demonstrated associations with liver steatosis in lean participants, but not with liver steatosis in overweight and obese participants. A history of ≥1 year exposure to INSTI in general, as well as the specific drug raltegravir, and non-INSTI drugs stavudine and IDV), were all associated with higher odds of liver steatosis in lean participants. The associations between history of exposure to these drugs with liver steatosis in lean PHIV are visualized in boxplots (Supplementary Figure 3), and the cumulative duration of exposure is depicted in scatter plots (Supplementary Figure 4). In contrast to the previously mentioned ART, current treatment with dual therapy was associated with decreased odds of liver steatosis in lean individuals.

#### Fibrosis

Apart from BMI and fat layer thickness, several other traditional factors were associated with liver fibrosis. T2DM and a history of myocardial infarction were strongly associated with increased odds for liver fibrosis in overweight/obese PHIV, whereas none of the assessed comorbidities was significantly associated with liver fibrosis in lean PHIV.

In addition, several laboratory measurements were associated with liver fibrosis. Decreased total, HDL, LDL, and very LDL cholesterol levels were associated with liver fibrosis with similar trends across subgroups. In addition, increased (nonfasting) glucose and decreased lactate dehydrogenase were associated with liver fibrosis in overweight/obese PHIV specifically.

In contrast to these traditional risk factors, none of the HIV-specific factors nor exposure to different antiretroviral drugs were associated with liver fibrosis.

#### Multivariate Logistic Regression

In the final multivariate models on liver steatosis, increased BMI, triglycerides, age, CD4 count at study enrollment, and prior infection remained associated with liver steatosis in the total population, whereas CD4 count at study enrollment and exposure to raltegravir remained associated with liver steatosis in lean PHIV (Supplementary Figure 5 and Supplementary Table 6).

In the final multivariate models on liver fibrosis, elevated ALT remained associated with liver fibrosis across groups. In addition, low HDL and increased BMI remained associated with liver fibrosis in the total study population, low LDL remained associated in lean PHIV, and low HDL and lactate dehydrogenase remained associated in overweight/obese PHIV (Supplementary Figure 6 and Supplementary Table 7).

In summary, traditional risk factors encompassing demographic, metabolic comorbidities, liver enzymes, and lipid profile were associated with liver steatosis and fibrosis in both lean and overweight/obese PHIV. However, history of exposure to specific ART was associated with steatosis in lean participants, and not in overweight/obese participants.

### Stronger Correlations Between HIV and ART Variables With Metabolic Factors in Lean Compared to Overweight/Obese PHIV

**Figure 5.**
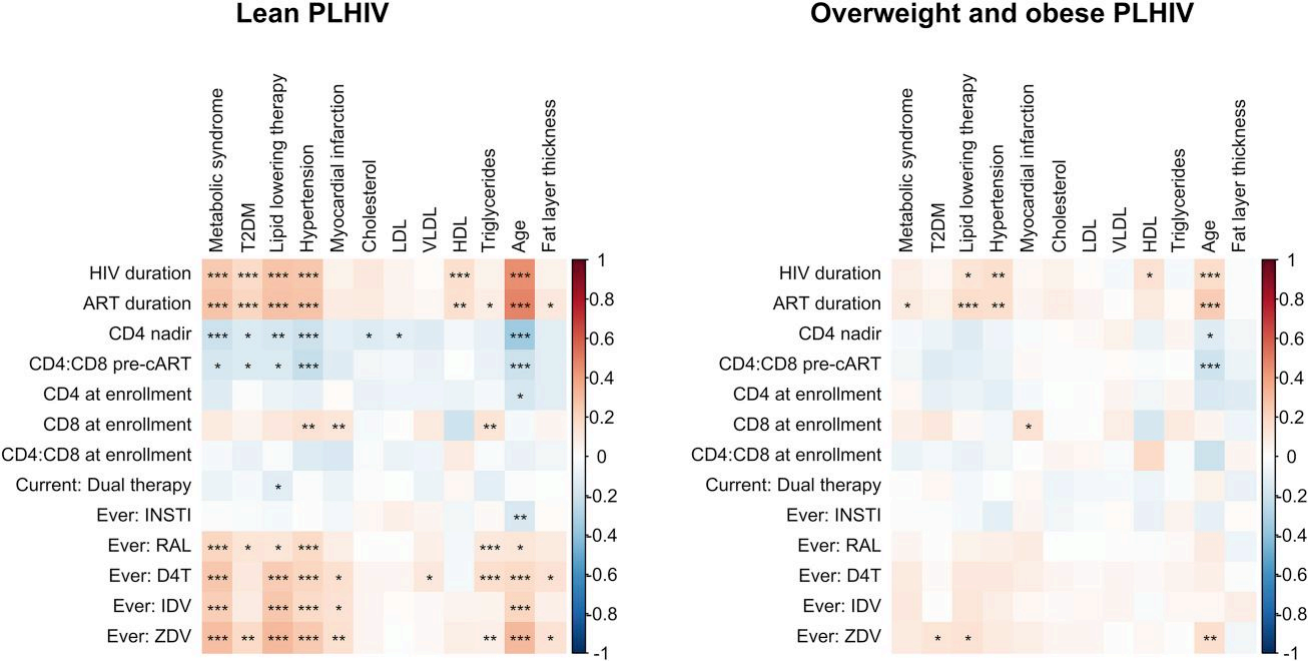
Correlations between HIV characteristics and duration of exposure to selected antiretrovirals with metabolic factors for lean (left) and overweight and obese PHIV (right). This correlation plot shows the correlations between HIV characteristics and ART exposure, with metabolic comorbidities and lipid measurements. Spearman's rho correlation coefficients were calculated for associations between continuous variables and Phi coefficients for associations between 2 binary variables (accompanied with the *P* value from Chi-square test). The color of the tiles shows the direction (red for positive, blue for negative) and strength of the association, the asterisks depict the level of significance of the FDR-corrected *P* values: *P values .01–.05; **P values .001–.01; and ***P values < .001. Abbreviations: ART, antiretroviral therapy; D4T, stavudine; HDL, high density lipoprotein; IDV, indinavir; INSTI, integrase strand transfer inhibitor; LDL, low-density lipoprotein; RAL, raltegravir; T2DM, type 2 diabetes mellitus; VLDL, very low-density lipoprotein; ZDV, zidovudine.

We correlated HIV- and ART-specific factors with several metabolic comorbidities and lipid profile. As expected, we found multiple significant correlations (eg, HIV and cART duration are positively correlated to metabolic syndrome, T2DM, and hypertension). On the other hand, CD4 nadir and CD4:CD8 ratio before cART initiation were negatively correlated with these traditional MASLD risk factors (Figure 5). Interestingly, these correlations were stronger and more frequently significant in lean PHIV compared to overweight/obese PHIV. In addition, the antiretrovirals that were significantly associated with liver steatosis in lean PHIV, were also found to be associated with metabolic syndrome, T2DM, LLT, and myocardial infarction in this subgroup. In overweight/obese PHIV, only minor correlations were found.

### Overweight and Obese PHIV With Liver Steatosis Have Reduced Self-reported Levels of Physical Activity

In addition, we assessed whether self-reported physical activity in general and during work is associated with steatosis and fibrosis in lean and overweight/obese PHIV. Among overweight/obese PHIV, those with steatosis reported lower levels of physical activity in general compared to those without steatosis (*P* < .001, Supplementary Figure 7). Similar trends were observed for lean PLHIV with and without liver steatosis, as well as for PHIV with and without fibrosis in both subgroups. Physical activity during work was significantly in lean PHIV with steatosis compared to those without, and a similar trend was seen in overweight/obese PHIV (Supplementary Figure 8).

## DISCUSSION

The prevalence and drivers of MASLD, formerly known as NAFLD, are well studied in the general population, but PHIV are often excluded from these studies. In addition, little is known regarding the etiology in lean PHIV. We assessed the prevalence and predictors of liver steatosis and fibrosis in both lean and overweight/obese PHIV on cART. Liver steatosis was highly prevalent in this European cohort, affecting 19.7% of lean PHIV, 54.0% of overweight/obese PHIV, and 37.7% of the total study population. Liver fibrosis was less common, affecting 5.9% of lean PHIV, 12.0% of overweight/obese PHIV, and 9.0% of the total population. Both liver steatosis and fibrosis were significantly associated with classic metabolic factors such as a dysregulated lipid profile and metabolic comorbidities. Interestingly, we found that a history of exposure to specific antiretroviral drugs predisposed lean PHIV to steatosis, whereas this association was not significant in overweight/obese PHIV. Notably, this entails mainly exposure to older antiretrovirals, including stavudine and indinavir, as well as exposure to INSTI, which are recommended in all current first-line regimens.

The reported prevalence's of liver steatosis and fibrosis in PHIV vary considerably, primarily because of the heterogeneity of the studied populations. A recent systematic review including 24 studies estimated a pooled steatosis prevalence of 38% (95% CI, 31-45) and a pooled fibrosis prevalence of 13% (95% CI, 8-18) in high- and middle-income countries, both with significant heterogeneity [27].

In few studies that assessed liver steatosis in lean PHIV, a prevalence of approximately one-third was estimated [12, 28]. We found a prevalence of one-fifth. However, when employing the same definition of liver steatosis (CAP value ≥248 dB/m), we found a similar prevalence of 29.8% (data not shown). Consequently, depending on the definition, liver steatosis affects approximately 20%–30% of lean PHIV, which is higher than prevalence estimates of lean NAFLD in the general European population (ranging from 8% to 20%) [29]. However, the prevalence of steatotic liver disease (SLD) may be as high as 64%–69.8% in PHIV at risk (defined as metabolic syndrome, elevated transaminases and/or lipodystrophy [30], or increased BMI [28]). Our data corroborate the differences in prevalence between different BMI groups. The prevalence of liver steatosis in overweight/obese PHIV was more than double compared to lean PHIV.

Importantly, increased BMI has also been associated with liver fibrosis. Indeed, we found that overweight/obese PHIV exhibited a more severe SLD phenotype compared to lean individuals, with a fibrosis prevalence of 16.7% in those with concomitant steatosis (compared to 8.0% of lean PHIV with concomitant steatosis). However, data on fibrosis prevalence among patients with SLD stratified by BMI category are scarce. Cervo et al. observed similar fibrosis prevalence for lean and overweight PHIV with NAFLD, but increased fibrosis prevalence amongst PHIV with obesity and NAFLD [12]. Another study assessed the probability of fibrosis using risk scores in PHIV with NAFLD and found no difference between lean and nonlean PHIV with NAFLD in likelihood of fibrosis [31]. Further studies are needed to assess the incidence of liver fibrosis in lean versus overweight and obese PHIV with MASLD.

The pathogenesis of MASLD is complex, multifactorial, and may differ in lean individuals. Traditionally, obesity, dyslipidemia, and T2DM are key determinants of liver steatosis and fibrosis [7, 32]. These traditional risk factors were also observed in PHIV (e.g., fat layer thickness was strongly associated with steatosis across subgroups, and the highest adjusted odds for fibrosis were found for fat layer thickness, T2DM, and prior history of myocardial infarction among overweight and obese PHIV). Another study performed in PHIV found that visceral fat was strongly associated with the presence and progression of fibrosis [33]. None of the traditional risk factors was associated with fibrosis in lean PHIV. However, the small sample size of lean PHIV with fibrosis (n = 7) limits our ability to draw conclusions from these results.

HIV infection itself, as well as specific antiretroviral drugs, may contribute to metabolic changes [34]. HIV viremia has been associated with dyslipidemia and insulin resistance [35], which can be attributed to immune activation and interaction with sterol regulatory element-binding-protein 1, peroxisome proliferator-activated receptor γ, and glucocorticoid receptors. These play crucial roles in the regulation of lipogenesis and insulin signaling [13, 36], potentially amplifying steatogenesis and fibrogenesis.

Previous and current ART regimens may also play a role in the development of liver steatosis in PHIV. We found that exposure to specific antiretroviral drugs is associated with liver steatosis in lean, but not in overweight/obese PHIV. Prior exposure to d-drugs [12, 28] and INSTI [12] were previously found to be associated with liver steatosis in lean PHIV. Here, exposure to stavudine, indinavir, INSTI in general, and raltegravir, were all identified as predictors of liver steatosis in lean PHIV. Interestingly, these concern mostly prior exposure because most participants discontinued treatment with these older drugs. It is well known that thymidine analogues (ie, stavudine and zidovudine) may influence fat metabolism and distribution, and these effects may persist for almost a decade [37]. Thymidine analogues are known to bind to intra-mitochondrial polymerase γ. This process inhibits the replication of mitochondrial DNA and impairs mitochondrial function [38]. Abnormal mitochondrial function decreases beta-oxidation of fatty acids and induces fat accumulation, eventually leading to steatosis and fibrosis [6, 15]. In addition, stavudine and zidovudine may cause insulin resistance, as do protease inhibitors such as indinavir [38]. Insulin resistance drives liver steatosis in 2 ways: by stimulating the release of free fatty acids by adipose tissue and by inducing synthesis of free fatty acids by the liver [39].

More recently, the relationship between exposure to INSTI and liver steatosis has been explored, with suggestions that INSTI may contribute to liver steatosis through INSTI-induced weight gain [17, 40]. However, the association between INSTI and weight gain remains controversial, and weight loss in comparator groups should be considered. Nonetheless, the association between INSTI and liver steatosis is particularly relevant given the widespread use of INSTI among PHIV (56.2% in our study population).

In our study, we found an association between exposure to raltegravir and liver steatosis in lean PHIV, whereas no significant association was found with other INSTI. In contrast, current treatment with dual therapy, which most often involves dolutegravir (3TC/DTG), was associated with lower odds of steatosis in lean individuals. A recent study also observed a significant association between raltegravir and liver steatosis, but not with dolutegravir [40]. Raltegravir leads to increased waist circumference compared to other INSTI [40, 41]. It is tempting to speculate why we found significant effects of raltegravir exposure solely in lean individuals. It could be possible that our lean group did in fact increase in weight and circumference but remained within the lean category. However, it is important to consider the potential presence of selection bias in the group of patients exposed to raltegravir because previous ART regimens may have led to an overestimation of MASLD in raltegravir-treated patients [42]. Additionally, raltegravir may have been the preferred antiretroviral drug for patients with multiple comorbidities because of limited drug-drug interaction [43]. Future studies should further explore the role of INSTI in general as well as raltegravir specifically in the development of liver steatosis.

The effects of ART exposure not only seem to be stronger for liver steatosis but also for other metabolic disorders in lean PHIV. HIV duration and history of treatment with specific ART including raltegravir, stavudine, and indinavir, were strongly correlated with metabolic disorders such as T2DM and hypertension. That these factors appear more important in lean compared to overweight/obese PHIV is striking. BMI may have such a strong impact on the occurrence of metabolic disorders in overweight/obese PHIV, that HIV-specific characteristics and ART exposure are overshadowed. In contrast, the metabolic effects of HIV and specific antiretroviral drugs could be most distinct in lean PHIV.

Identification of MASLD in both lean and overweight/obese PHIV is important to prevent chronic liver disease and cardiovascular disease. Liver steatosis was underrecognized in our cohort, being documented in a minority of medical files. The European Aids Clinical Society guideline (not yet adjusted to the new nomenclature) recommends screening for NAFLD in PHIV with obesity, metabolic syndrome, persistent ALT elevation, and exposure to d-drugs [44]. Lean PHIV lack the traditional obese phenotype that pertains to screening, which may explain why the majority of our patients was not recognized having steatosis. Moreover, the recent change in nomenclature may further complicate the identification of MASLD in lean PHIV. To fulfill the criteria of MASLD, at least 1 of the 5 metabolic syndrome components should be present on top of liver steatosis [3]. However, liver steatosis in lean PHIV is not solely driven by traditional metabolic factors. Instead, our results highlight d-drugs as a risk factor and indicates that exposure to antiretroviral drugs other than d-drugs (ie, raltegravir and indinavir) increases risk for liver steatosis as well.

This study has several limitations. First, the cross-sectional design hinders us from drawing conclusions on causal effects and impedes evaluation of the dynamics of liver steatosis in PHIV. Second, we were not able to perform liver biopsies, which remains the golden standard for diagnosis of liver steatosis and fibrosis. Consequently, we do not have information on the grade of steatohepatitis, which includes both steatosis and inflammation, and assess the true risk of liver disease in PHIV. Instead, we rely on VCTE for the assessment of liver steatosis and fibrosis. VCTE requires an experienced operator for accurate assessments, especially in people with obesity [45]. Furthermore, thresholds of CAP and LSM depend on the etiology of liver steatosis and fibrosis, respectively [45]. Moreover, even for specific etiologies, including liver steatosis in PHIV, there is little consensus on the most accurate threshold [27]. Nevertheless, VCTE is a widely used, noninvasive, and validated technique [45].

Despite these limitations, our study contributes to improving the identification of liver steatosis in PHIV through a large study including well-characterized individuals with HIV-1 monoinfection on long-term suppressive cART. This has allowed us to draft an additional consideration to the European Aids Clinical Society guideline on screening for steatosis (see graphical abstract), which focuses special attention of any HIV-treating physician to lean PHIV. By timely screening and identification of steatosis, healthcare providers can take proactive measures such as lifestyle adjustments and tailored cART to reduce the risk of liver disease.

In conclusion, liver steatosis is highly prevalent in PHIV affecting nearly one-fifth of lean and more than half of overweight/obese patients. In lean PHIV, not only traditional metabolic risk factors, but also exposure to specific ART, including INSTI, was associated with steatosis. In contrast, steatosis in overweight/obese PHIV seemed to be mainly associated with traditional risk factors. Diagnosing steatosis in lean PHIV remains challenging. Increasing awareness and screening of lean PHIV may lead to early identification of liver steatosis, ultimately reducing the burden of disease and improving the overall quality of care for PHIV.

## Supplementary Material

ofae266_Supplementary_Data

## Supplementary Data

Supplementary materials are available at *Open Forum Infectious Diseases* online. Consisting of data provided by the authors to benefit the reader, the posted materials are not copyedited and are the sole responsibility of the authors, so questions or comments should be addressed to the corresponding author.

## Notes

***Acknowledgments.*** The authors thank all participants of the 2000HIV study for their participation.

***Author Contributions.*** L. E. v. E., Q. d. M., L. A. B. J., and E. T. T. L. T. designed the study. L. E. v. E., W. A. J. W. V., M. J. T. B., and A. L. G. collected the data. L. E. v. E. and N. V. analyzed the data. L. E. v. E., Q. d. M., L. A. B. J., and E. T. T. L. T. interpreted the data. L. E. v. E. drafted the manuscript. N .V., W. A. J. W. V., M. J. T. B., A. L. G., J. v. L., J. S., M. J. T. B., A. V., G. W., M. G. N., A. J. A. M. v. d. V., Q. d. M., L. A. B. J., and E. T. T. L. T. critically revised the article.

***Financial support*.** The 2000HIV study is financially supported by ViiV Healthcare (A18-1052), which was awarded to AJAMvdV, MGN, LABJ, and QdM. ViiV Healthcare was not involved in the study design, data collection, data analysis, data interpretation, manuscript writing, nor in the decision to submit this manuscript for publication.
