# Supplementary material for: Liver Steatosis is Prevalent in Lean People With HIV and Associated With Exposure to Antiretroviral Treatment—A Cross-sectional Study
Source: Open Forum Infect Dis. 2024 May 7;11(6):ofae266. doi: 10.1093/ofid/ofae266 (PMC11167668; doi:10.1093/ofid/ofae266)
Supplement: ofae266_Supplementary_Data [file ofae266_supplementary_data.docx]

**Supplementary methods, figures, and tables**

**Methods**

**Data extraction and questionnaires**

Demographic data, clinical data including history of HIV and comorbidities, current cART regimens, comedication, and biometric data were registered. Laboratory tests performed within 13 months prior to the study visit including glucose and liver function tests (aspartate aminotransferase [AST], alanine aminotransferase [ALT], alkaline phosphatase [ALP], gamma-glutamyl transferase [GGT], lactate dehydrogenase [LDH], and bilirubin) were also obtained from medical files. A lipid profile (including total cholesterol, high-density lipoprotein (HDL), low-density lipoprotein (LDL), and very low-density lipoprotein (VLDL) cholesterol, and triglycerides) was measured in blood collected during the study visit using a high-throughput nuclear magnetic resonance spectroscopy platform (Nightingale’s Biomarker Analysis Platform, Helsinki). Methodological details have previously been reviewed(1).

CART history, as well as CD4 (nadir, before cART initiation, and latest measurement at enrollment [within 13 months prior to study visit]) and CD8 counts (before cART initiation and at enrollment) and HIV-RNA viral loads (zenith and at enrollment) were obtained from the Stichting HIV Monitoring (SHM, a national HIV registry). Because of possible association with liver steatosis or fibrosis(2, 3), we selected dideoxynucleosides, protease inhibitors, and integrase inhibitors as drug classes, all drugs belonging to these classes, and zidovudine, tenofovir alafenamide (TAF), tenofovir disoproxil fumarate (TDF) and efavirenz (EFV) to be included in the analysis. Two variables were created for each of the drug variables: a binary variable (no prior exposure vs. at least one year exposure) and a continuous variable (cumulative duration of exposure of all exposed individuals).

Past hepatitis B infection was defined as presence of hepatitis B surface antibody (anti-HBs) combined with presence of hepatitis B core antibody (anti-HBc).

Self-reported physical activity was assessed by questionaries (Supplementary methods). All information collected from medical files, SHM, and the questionnaires was collected in electronic case report forms (CRF) in CastorEDC.

**CMV IgG titer measurement**

Serum CMV IgG titers were measured according to manufacturer’s protocol using commercially available enzyme-linked immunosorbent assay (ELISA; Genway Biotech; San Diego, CA, USA, Catalog number GWB-892399).

**Ultrasound examination of the liver**

We performed standard ultrasonography of the liver using a low-frequency abdominal probe. Fat layer thickness, defined as the total thickness of superficial tissue layers (including skin, muscle, subcutaneous and visceral fat) between the transducer and the liver capsule, was obtained at the level of the right liver lobe. We used the data on fat layer thickness for logistic regression analyses.

|  | **Variable** | **Unit** |
| --- | --- | --- |
| ***Demographics*** | **Sex** | Male vs. female |
|  | **Age** | Per decade |
|  | **Black ethnicity** | No vs. yes |
|  | **Hispanic ethnicity** | No vs. yes |
|  | **Asian ethnicity** | No vs. yes |
|  | **Native American ethnicity** | No vs. yes |
|  | **Current smoking** | No vs. yes |
|  | **BMI** | Per 1 kg/m^2^ |
|  | **Fat layer thickness** | ≤ 25 mm^2^ vs. > 25 mm^2^ |
|  | **CMV IgG serology** | Negative vs. positive |
|  |  |  |
| ***Comorbidities*** | **T2DM** | No vs. yes |
|  | **Lipid lowering therapy** | No vs. yes |
|  | **Hypertension** | No vs. yes |
|  | **Myocardial infarction** | No vs. yes |
|  | **Cholecystectomy** | No vs. yes |
|  | **Past HAV infection** | No vs. yes |
|  | **Past HBV infection** | No vs. yes |
|  | **Past HCV infection** | No vs. yes |
|  |  |  |
| ***Laboratory measurements*** | **Cholesterol** | mmol/l |
|  | **LDL** | mmol/l |
|  | **VLDL** | mmol/l |
|  | **HDL** | mmol/l |
|  | **Triglycerides** | mmol/l |
|  | **Glucose** | mmol/l |
|  | **AST** | Per 10 U/L |
|  | **ALT** | Per 10 U/L |
|  | **ALP** | Per 10 U/L |
|  | **GGT** | Per 10 U/L |
|  | **Bilirubin** | Per 10 U/L |
|  | **LDH** | Per 10 U/L |
|  |  |  |
| ***HIV-specific characteristics*** | **HIV duration** | Per year |
|  | **CD4 nadir** | Per 100 * 10^6^ cells/l |
|  | **CD4:CD8 pre-cART** | Ratio |
|  | **Viral load zenith** | Per 10,000 copies/mL |
|  | **AIDS-defining wasting** | No vs. yes |
|  | **WOT: MSM** | No vs. yes |
|  | **WOT: blood products** | No vs. yes |
|  | **WOT: heterosexual transmission** | No vs. yes |
|  | **Residual viremia** | No vs. yes |
|  | **CD4 at inclusion** | Per 100 * 10^6^ cells/l |
|  | **CD8 at inclusion** | Per 100 * 10^6^ cells/l |
|  | **CD4:CD8 at inclusion** | Ratio |
|  |  |  |
| ***Exposure to ART*** | **Duration of untreated infection** | Per year |
|  | **ART duration** | Per year |
|  | **Current: no ART** | No vs. yes |
|  | **Current: dual therapy** | No vs. yes |
|  | **Current: NRTI** | No vs. yes |
|  | **Current: NtRTI** | No vs. yes |
|  | **Current: NNRTI** | No vs. yes |
|  | **Current: PI** | No vs. yes |
|  | **Current: INSTI** | No vs. yes |
|  | **Ever: d-drugs** | Never vs. ≥ 1 year |
|  | **Cumulative: d-drugs** | Per year |
|  | **Ever: PI** | Never vs. ≥ 1 year |
|  | **Cumulative: PI** | Per year |
|  | **Ever: INSTI** | Never vs. ≥ 1 year |
|  | **Cumulative: INSTI** | Per year |
|  | **Ever: D4T** | Never vs. ≥ 1 year |
|  | **Cumulative: D4T** | Per year |
|  | **Ever: DDC** | Never vs. ≥ 1 year |
|  | **Cumulative: DDC** | Per year |
|  | **Ever: DDI** | Never vs. ≥ 1 year |
|  | **Cumulative: DDI** | Per year |
|  | **Ever: IDV** | Never vs. ≥ 1 year |
|  | **Cumulative: IDV** | Per year |
|  | **Ever: RTV** | Never vs. ≥ 1 year |
|  | **Cumulative: RTV** | Per year |
|  | **Ever: ZDV** | Never vs. ≥ 1 year |
|  | **Cumulative: ZDV** | Per year |
|  | **Ever: RAL** | Never vs. ≥ 1 year |
|  | **Cumulative: RAL** | Per year |
|  | **Ever: BIC** | Never vs. ≥ 1 year |
|  | **Cumulative: BIC** | Per year |
|  | **Ever: DTG** | Never vs. ≥ 1 year |
|  | **Cumulative: DTG** | Per year |
|  | **Ever: EVG** | Never vs. ≥ 1 year |
|  | **Cumulative: EVG** | Per year |
|  | **Ever: DTG** | Never vs. ≥ 1 year |
|  | **Cumulative: DTG** | Per year |
|  | **Ever: TAF** | Never vs. ≥ 1 year |
|  | **Cumulative: TAF** | Per year |
|  | **Ever: TDF** | Never vs. ≥ 1 year |
|  | **Cumulative: TDF** | Per year |
|  | **Ever: EFV** | Never vs. ≥ 1 year |
|  | **Cumulative: EFV** | Per year |

**Supplementary Table 1.** Variables tested in logistic regression models. Abbreviations: CMV = cytomegalovirus, T2DM = type 2 diabetes mellitus, HAV = hepatitis A virus, HBV = hepatitis B virus, HCV = hepatitis C virus, HDL = high density lipoprotein, LDL = low density lipoprotein, VLDL = very low density lipoprotein, ALT = alanine transaminase, AST = aspartate aminotransferase, ALP = alkaline phosphatase, GGT = gamma-glutamyl transferase, LDH = lactate dehydrogenase, WOT = way of transmission, MSM = men who have sex with men, ART = antiretroviral therapy, NRTI = nucleoside reverse transcriptase inhibitor, NtRTI = nucleotide reverse transcriptase inhibitor, NNRTI = non-nucleoside reverse transcriptase inhibitor, PI = protease inhibitor, INSTI = integrase strand transfer inhibitor, d-drugs = dideoxynucleoside analogs, D4T = stavudine, DDC = zalcitabine, DDI = didanosine, IDV = indinavir, RTV = ritonavir, ZDV = zidovudine, RAL = raltegravir, BIC = bictegravir, DTG = dolutegravir, TAF = tenofovir alafenamide, TDF = tenofovir disoproxil fumarate, EFV = efavirenz.

|  |  | **BMI Classification** | |  |
| --- | --- | --- | --- | --- |
| **Variable** | **Overall**, N = 1,050*^1^* | **Lean**, N = 505*^1^* | **Overweight or obese**, N = 545*^1^* | **p-value***^2^* |
| **Ever: PI** | 289 (31%) | 145 (32%) | 144 (30%) | 0.43 |
| **Duration: PI** | 3.3 (1.2, 8.8) | 3.3 (1.2, 8.9) | 3.4 (1.2, 8.4) | 0.93 |
| **Ever: D-drugs** | 85 (8.4%) | 46 (9.5%) | 39 (7.4%) | 0.25 |
| **Duration: D-drugs** | 4.3 (2.1, 8.5) | 6.1 (3.2, 8.9) | 3.1 (1.6, 7.0) | **0.016** |
| **Ever: INSTI** | 582 (63%) | 269 (60%) | 313 (66%) | 0.10 |
| **Duration: INSTI** | 3.65 (1.82, 5.05) | 3.79 (1.82, 5.01) | 3.55 (1.82, 5.07) | 0.70 |
| **Ever: D4T** | 68 (6.8%) | 35 (7.2%) | 33 (6.3%) | 0.56 |
| **Duration: D4T** | 3.30 (1.61, 5.57) | 4.31 (1.79, 6.76) | 2.31 (1.54, 4.51) | 0.068 |
| **Ever: DDC** | 9 (0.9%) | 5 (1.1%) | 4 (0.8%) | 0.74 |
| **Duration: DDC** | 0.84 (0.43, 2.37) | 0.72 (0.42, 1.09) | 2.56 (1.29, 3.27) | 0.12 |
| **Ever: DDI** | 38 (3.8%) | 24 (5.0%) | 14 (2.7%) | 0.056 |
| **Duration: DDI** | 3.3 (1.1, 7.8) | 3.9 (1.3, 7.6) | 1.5 (0.9, 7.8) | 0.49 |
| **Ever: IDV** | 42 (4.2%) | 27 (5.6%) | 15 (2.9%) | **0.029** |
| **Duration: IDV** | 1.84 (0.98, 3.34) | 2.66 (1.17, 4.23) | 1.43 (0.81, 2.64) | 0.12 |
| **Ever: RTV** | 224 (24%) | 108 (24%) | 116 (24%) | 0.87 |
| **Duration: RTV** | 3.2 (0.8, 8.3) | 3.1 (0.7, 9.0) | 3.5 (0.9, 7.8) | 0.76 |
| **Ever: TAF** | 341 (36%) | 156 (34%) | 185 (37%) | 0.26 |
| **Duration: TAF** | 2.37 (1.55, 3.29) | 2.41 (1.55, 3.27) | 2.30 (1.56, 3.30) | 0.73 |
| **Ever: TDF** | 715 (76%) | 350 (77%) | 365 (75%) | 0.59 |
| **Duration: TDF** | 6.4 (3.3, 9.5) | 6.4 (3.6, 9.6) | 6.3 (3.0, 9.5) | 0.63 |
| **Ever: ZDV** | 190 (20%) | 98 (21%) | 92 (18%) | 0.36 |
| **Duration: ZDV** | 6.0 (1.4, 10.4) | 6.0 (1.8, 11.6) | 6.0 (1.2, 9.8) | 0.23 |
| **Ever: RAL** | 59 (6.0%) | 30 (6.2%) | 29 (5.7%) | 0.73 |
| **Duration: RAL** | 2.03 (0.68, 5.87) | 2.20 (1.19, 5.25) | 1.83 (0.48, 6.16) | 0.38 |
| **Ever: BIC** | 54 (5.5%) | 23 (4.9%) | 31 (6.1%) | 0.39 |
| **Duration: BIC** | 1.10 (0.61, 1.64) | 1.10 (0.61, 1.57) | 1.10 (0.61, 1.77) | 0.59 |
| **Ever: DTG** | 393 (43%) | 179 (41%) | 214 (45%) | 0.20 |
| **Duration: DTG** | 2.91 (1.25, 4.55) | 3.17 (1.19, 4.50) | 2.65 (1.26, 4.61) | 0.83 |
| **Ever: EVG** | 151 (15%) | 71 (15%) | 80 (15%) | 0.80 |
| **Duration: EVG** | 3.35 (2.23, 4.78) | 3.28 (2.10, 5.20) | 3.60 (2.35, 4.54) | 0.86 |
| **Ever: EFV** | 264 (28%) | 135 (30%) | 129 (27%) | 0.26 |
| **Duration: EFV** | 4.4 (1.1, 8.3) | 4.5 (1.2, 8.3) | 4.0 (1.0, 8.3) | 0.84 |
| *^1^*Median (IQR) or Frequency (%) | | | | |
| *^2^*Pearson's Chi-squared test; Wilcoxon rank sum test; Fisher's exact test; Wilcoxon rank sum exact test | | | | |

**Supplementary Table 2.** Baseline characteristics: ART history. In this table the ART history is shown for the total group of participants with a valid CAP or LSM result, as well as for the subgroups “lean” and “overweight/obese”. Dichotomous variables are shown as number (%), and numeric variables are shown as median [Q1 – Q3]. For variables on (past) exposure to specific antiretrovirals: all values of at least 1 month exposure are taken into account here. The column P-value depicts the p-values of comparisons (Chi-Square test for dichotomous variables and Mann-Whitney U for numeric variables) between lean and overweight/obese participants. P-values ≥ 0.05 are depicted as “NS”. Abbreviations: PI = protease inhibitor, d-drugs = dideoxynucleoside analogs, INSTI = integrase strand transfer inhibitor, D4T = stavudine, DDC = zalcitabine, DDI = didanosine, IDV = indinavir, RTV = ritonavir, TAF = tenofovir alafenamide, TDF = tenofovir disoproxil fumarate, ZDV = zidovudine, RAL = raltegravir, BIC = bictegravir, DTG = dolutegravir, EVG = elvitegravir, EFV = efavirenz.

**
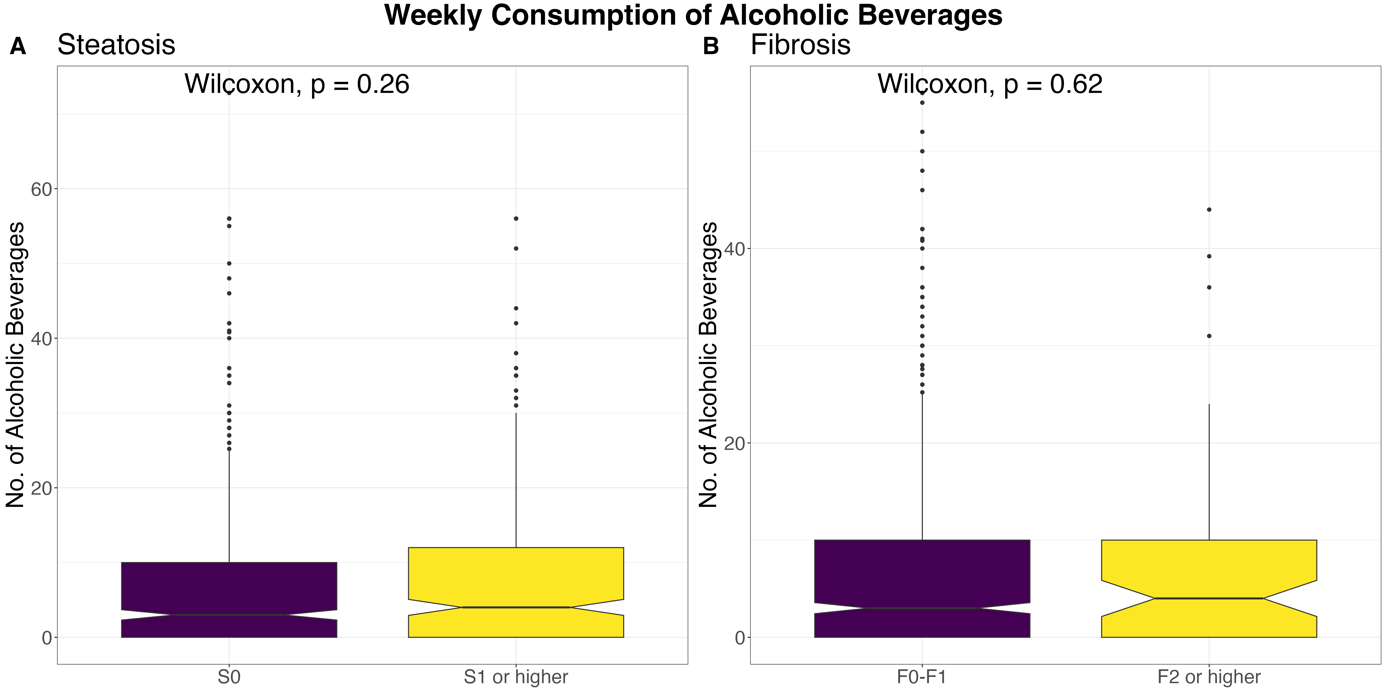
**

**Supplementary Figure 1.** Notched boxplots comparing weekly consumption of alcohol beverages between PLHIV with and without steatosis (A) and PLHIV with and without fibrosis (B) by Wilcoxon rank-sum test.


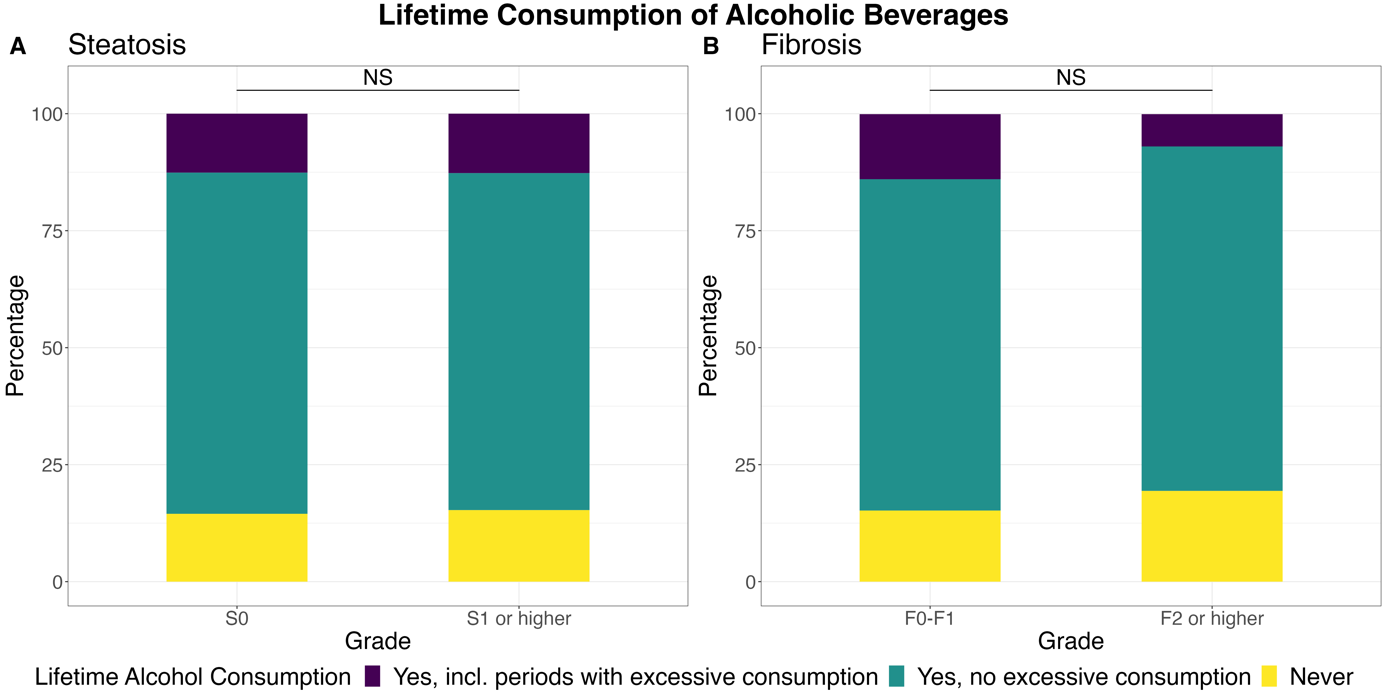


**Supplementary Figure 2.** Barplots showing the lifetime exposure of alcohol consumption between PLHIV with and without steatosis (A) and PLHIV with and without fibrosis (B) by chi-squared tests. Excessive alcohol consumption was defined as more than 28 (men) or 21 (women) alcohol beverages per week.

|  |  | | BMI classification | | | |
| --- | --- | --- | --- | --- | --- | --- |
|  | **Overall** | | **Lean PLHIV** | | **Overweight or obese PLHIV** | |
|  | **OR** | **P-value** | **OR** | **P-value** | **OR** | **P-value** |
| Female sex | 0.72 [0.48-1.07] | NS | 0.8 [0.34-1.86] | NS | 0.47 [0.29-0.75] | 0.006 |
| Age | 1.54 [1.36-1.73] | < 0.001 | 1.78 [1.44-2.2] | < 0.001 | 1.42 [1.21-1.67] | < 0.001 |
| Black ethnicity | 0.58 [0.38-0.89] | 0.040 | 0.37 [0.11-1.24] | NS | 0.38 [0.24-0.62] | < 0.001 |
| Hispanic ethnicity | 1.03 [0.53-2] | NS | 1.59 [0.55-4.58] | NS | 0.77 [0.32-1.84] | NS |
| Asian ethnicity | 0.74 [0.43-1.26] | NS | 0.37 [0.09-1.62] | NS | 0.62 [0.33-1.16] | NS |
| Current smoking | 0.75 [0.56-1] | NS | 0.89 [0.55-1.44] | NS | 0.86 [0.58-1.28] | NS |
| BMI | 1.27 [1.22-1.32] | < 0.001 | 1.41 [1.21-1.64] | < 0.001 | 1.18 [1.1-1.25] | < 0.001 |
| Fatlayer thickness | 5.36 [4.02-7.16] | < 0.001 | 3.78 [2.23-6.41] | < 0.001 | 3.12 [2.09-4.66] | < 0.001 |
| CMV IgG serology | 1.07 [0.63-1.79] | NS | 0.89 [0.39-2.01] | NS | 0.96 [0.45-2.03] | NS |
| T2DM | 2.3 [1.21-4.4] | 0.011 | 3.03 [1.02-8.96] | 0.046 | 1.72 [0.78-3.79] | NS |
| Lipid lowering therapy | 1.37 [0.95-1.98] | NS | 3.77 [2.03-6.98] | < 0.001 | 0.78 [0.49-1.23] | NS |
| Hypertension | 1.23 [0.88-1.74] | NS | 1.5 [0.82-2.76] | NS | 0.98 [0.65-1.49] | NS |
| Myocardial infarction | 1.71 [0.8-3.66] | NS | 4.66 [1.55-14.03] | 0.006 | 0.79 [0.29-2.15] | NS |
| Cholecystectomy | 0.67 [0.27-1.65] | NS | 0.88 [0.16-4.83] | NS | 0.62 [0.21-1.81] | NS |
| Past HAV infection | 1.09 [0.67-1.76] | NS | 0.83 [0.36-1.94] | NS | 1.23 [0.66-2.29] | NS |
| Past HBV infection | 0.7 [0.51-0.97] | 0.034 | 0.47 [0.25-0.88] | 0.018 | 0.74 [0.5-1.11] | NS |
| Past HCV infection | 0.65 [0.37-1.15] | NS | 1.01 [0.42-2.44] | NS | 0.52 [0.25-1.09] | NS |
| Cholesterol | 1.15 [0.99-1.33] | NS | 0.88 [0.67-1.16] | NS | 1.29 [1.07-1.56] | 0.009 |
| LDL | 1.43 [1.05-1.95] | 0.023 | 0.79 [0.45-1.39] | NS | 1.83 [1.23-2.73] | 0.003 |
| VLDL | 5.12 [3.01-8.72] | < 0.001 | 2.54 [0.99-6.51] | NS | 6.7 [3.32-13.54] | < 0.001 |
| HDL | 0.38 [0.24-0.63] | < 0.001 | 0.45 [0.2-1.05] | NS | 0.4 [0.21-0.74] | 0.004 |
| Triglycerides | 2.47 [1.95-3.12] | < 0.001 | 2.34 [1.54-3.55] | < 0.001 | 2.35 [1.75-3.16] | < 0.001 |
| Glucose | 1.24 [1.08-1.43] | 0.003 | 1.12 [0.85-1.47] | NS | 1.25 [1.04-1.49] | 0.015 |
| AST | 1.23 [0.92-1.63] | NS | 1.2 [0.73-1.96] | NS | 1.32 [0.86-2.03] | NS |
| ALT | 1.19 [1.09-1.3] | < 0.001 | 1.14 [1.02-1.29] | 0.026 | 1.2 [1.06-1.35] | 0.003 |
| ALP | 0.98 [0.91-1.06] | NS | 0.96 [0.84-1.1] | NS | 1.03 [0.91-1.16] | NS |
| GGT | 1.02 [0.98-1.07] | NS | 1.04 [0.97-1.11] | NS | 1.02 [0.97-1.07] | NS |
| Bilirubin | 1 [0.74-1.34] | NS | 0.66 [0.26-1.67] | NS | 1.1 [0.76-1.59] | NS |
| LDH | 1.02 [0.96-1.08] | NS | 1.02 [0.92-1.13] | NS | 0.99 [0.93-1.07] | NS |
| HIV duration | 1 [0.98-1.02] | NS | 1.02 [0.98-1.06] | NS | 0.98 [0.96-1.01] | NS |
| CD4 nadir | 0.99 [0.92-1.07] | NS | 0.97 [0.84-1.12] | NS | 0.99 [0.91-1.08] | NS |
| CD4:CD8 pre-cART | 0.95 [0.51-1.77] | NS | 0.39 [0.11-1.4] | NS | 1.31 [0.62-2.79] | NS |
| Viral load zenith | 1 [1-1] | NS | 1 [1-1] | NS | 1 [1-1] | NS |
| AIDS-defining wasting | 1.01 [0.63-1.63] | NS | 0.84 [0.35-2.01] | NS | 1.09 [0.6-1.99] | NS |
| WOT: MSM | 0.97 [0.7-1.34] | NS | 0.71 [0.39-1.27] | NS | 1.31 [0.88-1.94] | NS |
| WOT: IV drugs | 0.21 [0.02-1.84] | NS | 0 [0-Inf] | NS | 0.23 [0.03-2.05] | NS |
| WOT: blood products | 0.86 [0.1-7.75] | NS | 1607680.92 [0-Inf] | NS | 0.38 [0.03-4.8] | NS |
| WOT: congenital | 0 [0-Inf] | NS | 0 [0-Inf] | NS | 0 [0-Inf] | NS |
| WOT: heterosexual | 1.17 [0.83-1.64] | NS | 1.47 [0.79-2.75] | NS | 0.89 [0.59-1.33] | NS |
| Residual viremia | 0.71 [0.5-1] | NS | 0.75 [0.41-1.35] | NS | 0.74 [0.47-1.14] | NS |
| CD4 at enrollment | 1.12 [1.05-1.2] | 0.001 | 1.18 [1.05-1.33] | 0.004 | 1.08 [0.98-1.18] | NS |
| CD8 at enrollment | 1.04 [1-1.08] | 0.039 | 1.07 [1.01-1.13] | 0.032 | 1.02 [0.96-1.07] | NS |
| CD4:CD8 at enrollment | 1.25 [0.73-2.14] | NS | 2.18 [0.91-5.27] | NS | 1 [0.49-2.03] | NS |
| Duration of untreated infection | 1 [0.95-1.06] | NS | 0.98 [0.87-1.1] | NS | 1 [0.93-1.06] | NS |
| ART duration | 1 [0.98-1.03] | NS | 1.04 [1-1.09] | 0.039 | 0.99 [0.96-1.02] | NS |
| Current: no cART | 0.86 [0.23-3.15] | NS | 1.27 [0.09-18.62] | NS | 0.77 [0.17-3.56] | NS |
| Current: duotherapy | 0.94 [0.61-1.45] | NS | 0.29 [0.1-0.86] | 0.026 | 1.16 [0.69-1.94] | NS |
| Current: NRTI | 0.9 [0.44-1.85] | NS | 0.62 [0.21-1.87] | NS | 0.94 [0.37-2.43] | NS |
| Current: NtRTI | 1.15 [0.85-1.56] | NS | 1.49 [0.86-2.58] | NS | 1.05 [0.72-1.54] | NS |
| Current: NNRTI | 0.81 [0.6-1.1] | NS | 0.82 [0.48-1.38] | NS | 0.85 [0.57-1.26] | NS |
| Current: PI | 1.15 [0.7-1.9] | NS | 0.93 [0.4-2.17] | NS | 1.33 [0.7-2.54] | NS |
| Current: INSTI | 1.13 [0.84-1.53] | NS | 1.33 [0.79-2.23] | NS | 1.03 [0.71-1.51] | NS |
| Ever: d-drugs | 1.28 [0.76-2.16] | NS | 2.46 [1.19-5.07] | 0.015 | 0.87 [0.42-1.83] | NS |
| Cumulative: d-drugs | 1.06 [0.97-1.17] | NS | 1.12 [0.96-1.3] | NS | 1.05 [0.92-1.19] | NS |
| Ever: PI | 1.09 [0.78-1.52] | NS | 1.19 [0.67-2.1] | NS | 1.15 [0.74-1.77] | NS |
| Cumulative: PI | 1 [0.96-1.04] | NS | 1 [0.94-1.07] | NS | 1 [0.95-1.05] | NS |
| Ever: INSTI | 1.26 [0.91-1.74] | NS | 2 [1.12-3.58] | 0.019 | 0.96 [0.64-1.46] | NS |
| Cumulative: INSTI | 1.07 [0.99-1.16] | NS | 1.12 [0.98-1.27] | NS | 1.05 [0.95-1.16] | NS |
| Ever: D4T | 1.61 [0.9-2.87] | NS | 3.73 [1.69-8.2] | 0.001 | 0.87 [0.39-1.94] | NS |
| Cumulative: D4T | 1.23 [1.01-1.5] | 0.043 | 1.33 [1-1.77] | NS | 1.18 [0.88-1.58] | NS |
| Ever: DDC | 1.04 [0.24-4.55] | NS | 0.86 [0.08-8.82] | NS | 1.61 [0.17-15.74] | NS |
| Cumulative: DDC | 0.85 [0.46-1.59] | NS | 0.42 [0.05-3.45] | NS | 0 [0-Inf] | NS |
| Ever: DDI | 1.21 [0.56-2.63] | NS | 2.2 [0.82-5.93] | NS | 0.86 [0.26-2.85] | NS |
| Cumulative: DDI | 1.05 [0.9-1.22] | NS | 1.11 [0.87-1.41] | NS | 0.99 [0.8-1.21] | NS |
| Ever: IDV | 1.02 [0.5-2.06] | NS | 3.86 [1.59-9.37] | 0.003 | 0.23 [0.07-0.75] | 0.015 |
| Cumulative: IDV | 0.93 [0.69-1.26] | NS | 1 [0.71-1.42] | NS | 0.67 [0.3-1.48] | NS |
| Ever: RTV | 1.07 [0.74-1.54] | NS | 0.78 [0.41-1.47] | NS | 1.43 [0.88-2.3] | NS |
| Cumulative: RTV | 0.98 [0.94-1.03] | NS | 1 [0.93-1.08] | NS | 0.99 [0.92-1.06] | NS |
| Ever: ZDV | 1.02 [0.7-1.51] | NS | 1.75 [0.96-3.2] | NS | 0.77 [0.47-1.28] | NS |
| Cumulative: ZDV | 1.03 [0.98-1.09] | NS | 1 [0.92-1.07] | NS | 1.07 [0.99-1.17] | NS |
| Ever: RAL | 1.93 [1.04-3.59] | 0.038 | 3.6 [1.53-8.47] | 0.003 | 1.16 [0.5-2.71] | NS |
| Cumulative: RAL | 1.09 [0.94-1.27] | NS | 1.09 [0.84-1.42] | NS | 1.11 [0.91-1.36] | NS |
| Ever: BIC | 0.62 [0.31-1.26] | NS | 0.79 [0.2-3.13] | NS | 0.51 [0.23-1.15] | NS |
| Cumulative: BIC | 0.59 [0.27-1.29] | NS | 0.85 [0.11-6.81] | NS | 0.51 [0.21-1.26] | NS |
| Ever: DTG | 0.9 [0.66-1.24] | NS | 1.19 [0.68-2.08] | NS | 0.72 [0.48-1.07] | NS |
| Cumulative: DTG | 1.02 [0.91-1.14] | NS | 1.07 [0.87-1.32] | NS | 1 [0.86-1.15] | NS |
| Ever: EVG | 1.45 [0.95-2.21] | NS | 1.98 [0.98-3.98] | NS | 1.36 [0.79-2.34] | NS |
| Cumulative: EVG | 1.26 [1.03-1.53] | 0.022 | 1.17 [0.88-1.56] | NS | 1.36 [1.02-1.82] | 0.036 |
| Ever: EFV | 0.75 [0.53-1.06] | NS | 0.77 [0.42-1.4] | NS | 0.77 [0.49-1.2] | NS |
| Cumulative: EFV | 0.98 [0.93-1.04] | NS | 1 [0.9-1.1] | NS | 0.97 [0.91-1.04] | NS |
| Ever: TAF | 1.01 [0.74-1.39] | NS | 1.22 [0.7-2.14] | NS | 0.93 [0.62-1.39] | NS |
| Cumulative: TAF | 1.01 [0.84-1.21] | NS | 0.92 [0.67-1.27] | NS | 1.08 [0.85-1.37] | NS |
| Ever: TDF | 0.89 [0.62-1.28] | NS | 0.8 [0.42-1.5] | NS | 1 [0.64-1.58] | NS |
| Cumulative: TDF | 1 [0.97-1.05] | NS | 1.02 [0.95-1.09] | NS | 1.01 [0.96-1.06] | NS |

**Supplementary Table 3.** Associations between steatosis and demographics, comorbidities, laboratory measurements, HIV-specific characteristics, and ART-exposure for the total population (columns “All”), lean participants (columns “Lean”) and overweight and obese participants (columns “Overweight and obese”). Effect estimates are presented as odds ratios with 95% confidence intervals. The odds ratios for demographics are not corrected for any confounders. Age and fat layer thickness were added to the logistic regression models with comorbidities, HIV-specific characteristics, and ART exposure. For the models with laboratory assessments, we also added lipid lowering therapy as a confounders. Abbreviations: CMV = cytomegalovirus, T2DM = type 2 diabetes mellitus, HAV = hepatitis A virus, HBV = hepatitis B virus, HCV = hepatitis C virus, HDL = high density lipoprotein, LDL = low density lipoprotein, VLDL = very low density lipoprotein, ALT = alanine transaminase, AST = aspartate aminotransferase, ALP = alkaline phosphatase, GGT = gamma-glutamyl transferase, LDH = lactate dehydrogenase, WOT = way of transmission, MSM = men who have sex with men, ART = antiretroviral therapy, NRTI = nucleoside reverse transcriptase inhibitor, NtRTI = nucleotide reverse transcriptase inhibitor, NNRTI = non-nucleoside reverse transcriptase inhibitor, PI = protease inhibitor, INSTI = integrase strand transfer inhibitor, d-drugs = dideoxynucleoside analogs, D4T = stavudine, DDC = zalcitabine, DDI = didanosine, IDV = indinavir, RTV = ritonavir, ZDV = zidovudine, RAL = raltegravir, BIC = bictegravir, DTG = dolutegravir, EVG = elvitegravir, EFV = efavirenz, TAF = tenofovir alafenamide, TDF = tenofovir disoproxil fumarate.

|  |  | | BMI classification | | | |
| --- | --- | --- | --- | --- | --- | --- |
|  | **Total population** | | **Lean** | | **Overweight or obese** | |
|  | **OR** | **P-value** | **OR** | **P-value** | **OR** | **P-value** |
| Age ≥ 30 | 3.9 [1.5-10.12] | 0.019 | 5.14 [0.68-38.81] | NS | 3.34 [1.05-10.62] | NS |
| Age ≥ 40 | 2.93 [1.97-4.38] | < 0.001 | 4.88 [2.07-11.53] | 0.002 | 2.1 [1.26-3.48] | 0.017 |
| Age ≥ 50 | 2.47 [1.87-3.25] | < 0.001 | 3.1 [1.85-5.18] | < 0.001 | 2.11 [1.47-3.04] | < 0.001 |
| Age ≥ 60 | 2.16 [1.61-2.9] | < 0.001 | 2.95 [1.81-4.81] | < 0.001 | 1.94 [1.29-2.93] | 0.007 |
| Age ≥ 70 | 1.64 [0.94-2.85] | NS | 2.73 [1.19-6.23] | 0.048 | 1.25 [0.57-2.76] | NS |
| Age ≥ 80 | 1.65 [0.1-26.54] | NS | 0 [0-Inf] | NS | 666301.42 [0-Inf] | NS |

**Supplementary Table 4.** Odds ratios for the associations between different age groups and steatosis for the total population (columns “All”), lean participants (columns “Lean”) and overweight and obese participants (columns “Overweight and obese”). Effect estimates are presented as odds ratios with 95% confidence intervals. The odds ratios are not corrected for any confounders.


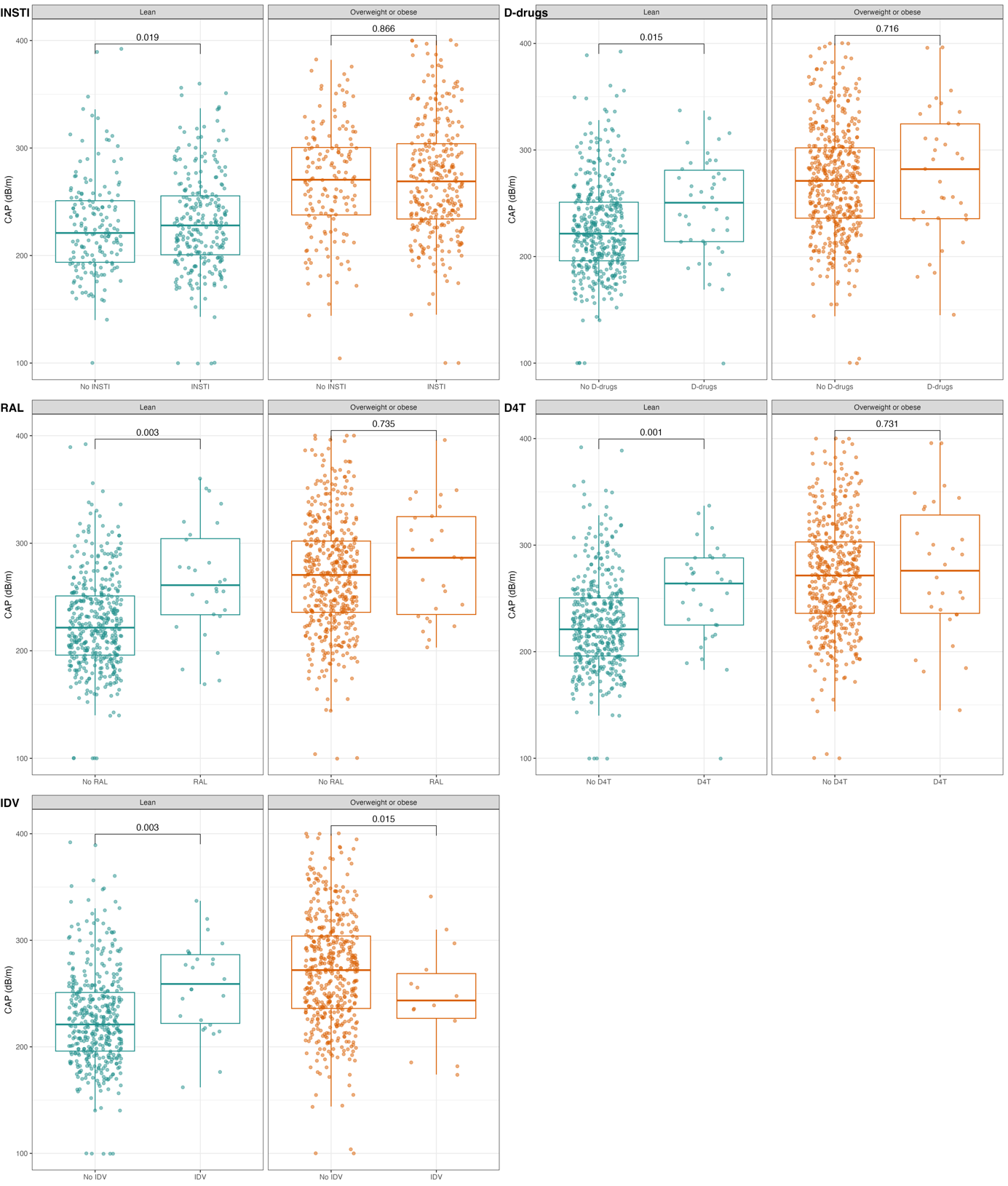


**Supplementary Figure 3.** Boxplots comparing the exposure to specific antiretrovirals (A: INSTI, B: raltegravir, C: stavudine, and D: indinavir) between participants with and without liver steatosis, in green for lean PLHIV and in orange for overweight and obese PLHIV. The p-values are derived from the logistic regression analysis: liver steatosis yes or no ~ antiretroviral + age + fat layer thickness. Abbreviations: INSTI = integrase strand transfer inhibitor, RAL = raltegravir, D4T = stavudine, IDV = indinavir, ZDV = zidovudine.


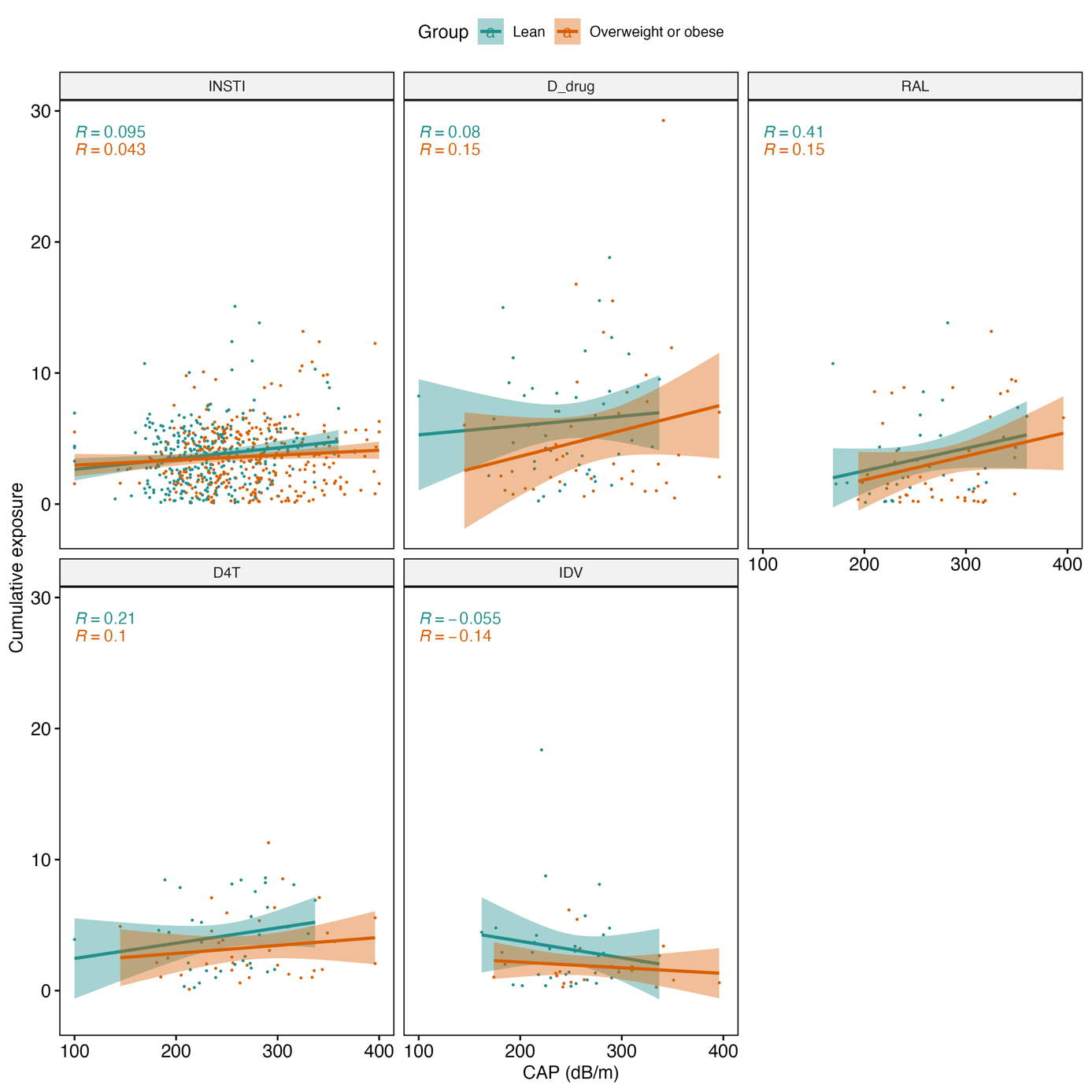


**Supplementary Figure 4.** Scatter plots showing the association between cumulative exposure of specific antiretrovirals (INSTI, RAL, D4T, and IDV) and CAP value, in green for lean PLHIV and in orange for overweight and obese PLHIV. Spearman correlation coefficients are shown separately for lean (in green) and for overweight and obese PLHIV (in orange). Abbreviations: RAL = raltegravir, D4T = stavudine, and IDV = indinavir. Abbreviations: INSTI = integrase strand transfer inhibitor, D_drug = dideoxynucleosides, RAL = raltegravir, D4T = stavudine, IDV = indinavir.

|  |  | | BMI classification | | | |
| --- | --- | --- | --- | --- | --- | --- |
|  | **Total population** | | **Lean** | | **Overweight or obese** | |
|  | **OR** | **P-value** | **OR** | **P-value** | **OR** | **P-value** |
| Female sex | 0.61 [0.29-1.29] | NS | 0.75 [0.17-3.25] | NS | 0.48 [0.2-1.15] | NS |
| Age | 1.12 [0.93-1.35] | NS | 0.93 [0.69-1.27] | NS | 1.21 [0.95-1.53] | NS |
| Black ethnicity | 0.47 [0.2-1.09] | NS | 0.83 [0.19-3.63] | NS | 0.31 [0.11-0.88] | NS |
| Hispanic ethnicity | 0.86 [0.26-2.84] | NS | 0 [0-Inf] | NS | 1.31 [0.37-4.59] | NS |
| Asian ethnicity | 0.97 [0.41-2.31] | NS | 0.63 [0.08-4.8] | NS | 0.99 [0.37-2.62] | NS |
| Current smoking | 0.97 [0.6-1.56] | NS | 1.22 [0.56-2.66] | NS | 0.97 [0.52-1.81] | NS |
| BMI | 1.14 [1.08-1.19] | < 0.001 | 1.01 [0.81-1.25] | NS | 1.16 [1.08-1.25] | < 0.001 |
| Fatlayer thickness | 2.78 [1.72-4.48] | < 0.001 | 0.99 [0.36-2.69] | NS | 4.33 [1.82-10.32] | 0.004 |
| CMV IgG serology | 1.53 [0.54-4.32] | NS | 7859626.66 [0-Inf] | NS | 0.81 [0.27-2.43] | NS |
| T2DM | 3.7 [1.82-7.53] | < 0.001 | 1.09 [0.13-8.93] | NS | 4.86 [2.17-10.9] | < 0.001 |
| Lipid lowering therapy | 1.49 [0.87-2.55] | NS | 1.13 [0.37-3.41] | NS | 1.63 [0.87-3.03] | NS |
| Hypertension | 1.01 [0.6-1.71] | NS | 1.07 [0.36-3.15] | NS | 0.96 [0.52-1.77] | NS |
| Myocardial infarction | 2.96 [1.2-7.31] | 0.019 | 2.56 [0.52-12.61] | NS | 4.47 [1.37-14.52] | 0.013 |
| Cholecystectomy | 0.76 [0.17-3.37] | NS | 0 [0-Inf] | NS | 0.92 [0.2-4.23] | NS |
| Past HAV infection | 1.06 [0.51-2.22] | NS | 1.83 [0.59-5.72] | NS | 0.75 [0.28-1.99] | NS |
| Past HBV infection | 0.8 [0.48-1.35] | NS | 0.63 [0.22-1.75] | NS | 0.83 [0.45-1.53] | NS |
| Past HCV infection | 0.88 [0.37-2.1] | NS | 0 [0-Inf] | NS | 1.64 [0.64-4.21] | NS |
| Cholesterol | 0.62 [0.48-0.79] | < 0.001 | 0.43 [0.27-0.69] | < 0.001 | 0.73 [0.54-0.97] | 0.032 |
| LDL | 0.44 [0.27-0.72] | 0.001 | 0.19 [0.07-0.5] | < 0.001 | 0.63 [0.35-1.12] | NS |
| VLDL | 0.74 [0.34-1.6] | NS | 0.18 [0.03-0.94] | 0.042 | 1.18 [0.5-2.81] | NS |
| HDL | 0.18 [0.07-0.45] | < 0.001 | 0.32 [0.08-1.23] | NS | 0.11 [0.03-0.39] | < 0.001 |
| Triglycerides | 1.14 [0.86-1.51] | NS | 0.78 [0.38-1.59] | NS | 1.24 [0.91-1.69] | NS |
| Glucose | 1.17 [1.02-1.34] | 0.024 | 0.79 [0.43-1.45] | NS | 1.22 [1.05-1.43] | 0.011 |
| AST | 1.18 [0.85-1.63] | NS | 1.1 [0.58-2.1] | NS | 1.15 [0.79-1.66] | NS |
| ALT | 1.19 [1.08-1.3] | < 0.001 | 1.17 [1.01-1.36] | 0.037 | 1.19 [1.06-1.34] | 0.002 |
| ALP | 1.05 [0.94-1.17] | NS | 1.17 [0.99-1.37] | NS | 1.02 [0.88-1.18] | NS |
| GGT | 1.03 [0.98-1.07] | NS | 0.98 [0.82-1.17] | NS | 1.03 [0.98-1.08] | NS |
| Bilirubin | 1.11 [0.79-1.56] | NS | 1.13 [0.57-2.23] | NS | 1.1 [0.75-1.63] | NS |
| LDH | 0.93 [0.86-1.01] | NS | 1.08 [0.94-1.22] | NS | 0.88 [0.79-0.98] | 0.015 |
| HIV duration | 0.97 [0.94-1.01] | NS | 0.98 [0.92-1.05] | NS | 0.98 [0.93-1.02] | NS |
| CD4 nadir | 1.01 [0.9-1.13] | NS | 1.01 [0.83-1.24] | NS | 0.99 [0.87-1.14] | NS |
| CD4:CD8 pre-cART | 0.77 [0.27-2.17] | NS | 0.38 [0.06-2.48] | NS | 1.04 [0.3-3.59] | NS |
| Viral load zenith | 1 [1-1] | 0.042 | 1 [1-1] | 0.044 | 1 [1-1] | NS |
| AIDS-defining wasting | 0.7 [0.31-1.58] | NS | 0.93 [0.21-4.16] | NS | 0.65 [0.24-1.71] | NS |
| WOT: MSM | 1.2 [0.72-2.01] | NS | 2.25 [0.66-7.67] | NS | 1.03 [0.57-1.86] | NS |
| WOT: IV drugs | 0 [0-Inf] | NS | 0 [0-Inf] | NS | 0 [0-Inf] | NS |
| WOT: blood products | 2.58 [0.25-26.21] | NS | 0 [0-Inf] | NS | 4.09 [0.32-51.86] | NS |
| WOT: congenital | 0 [0-Inf] | NS | 0 [0-Inf] | NS | 0 [0-Inf] | NS |
| WOT: heterosexual | 0.68 [0.39-1.2] | NS | 0.87 [0.29-2.6] | NS | 0.61 [0.32-1.18] | NS |
| Residual viremia | 1.01 [0.58-1.74] | NS | 1.06 [0.4-2.77] | NS | 1.04 [0.54-2.03] | NS |
| CD4 at enrollment | 0.99 [0.89-1.1] | NS | 0.97 [0.8-1.17] | NS | 0.99 [0.87-1.12] | NS |
| CD8 at enrollment | 1.01 [0.95-1.08] | NS | 1 [0.89-1.11] | NS | 1.02 [0.94-1.11] | NS |
| CD4:CD8 at enrollment | 0.48 [0.17-1.32] | NS | 0.79 [0.19-3.31] | NS | 0.3 [0.07-1.34] | NS |
| Duration of untreated infection | 0.96 [0.87-1.05] | NS | 1.08 [0.95-1.24] | NS | 0.87 [0.74-1.03] | NS |
| ART duration | 0.98 [0.94-1.02] | NS | 1 [0.93-1.07] | NS | 0.98 [0.94-1.03] | NS |
| Current: no cART | 0.76 [0.1-6.06] | NS | 4.3 [0.46-40.45] | NS | 0 [0-Inf] | NS |
| Current: duotherapy | 1.09 [0.57-2.1] | NS | 1.09 [0.31-3.78] | NS | 1.09 [0.5-2.37] | NS |
| Current: NRTI | 1.47 [0.44-4.94] | NS | 0.52 [0.11-2.43] | NS | 3.01 [0.39-23.08] | NS |
| Current: NtRTI | 1.1 [0.69-1.77] | NS | 0.5 [0.23-1.11] | NS | 1.64 [0.9-3.01] | NS |
| Current: NNRTI | 0.84 [0.52-1.37] | NS | 0.81 [0.35-1.88] | NS | 0.86 [0.47-1.57] | NS |
| Current: PI | 0.72 [0.3-1.71] | NS | 0.41 [0.05-3.1] | NS | 0.95 [0.36-2.56] | NS |
| Current: INSTI | 1.31 [0.81-2.09] | NS | 1.28 [0.56-2.9] | NS | 1.34 [0.75-2.4] | NS |
| Ever: d-drugs | 1.38 [0.65-2.94] | NS | 1.35 [0.37-4.96] | NS | 1.84 [0.7-4.82] | NS |
| Ever: PI | 1.22 [0.74-2.03] | NS | 0.87 [0.32-2.39] | NS | 1.64 [0.9-3] | NS |
| Ever: INSTI | 1.36 [0.81-2.28] | NS | 1.05 [0.44-2.49] | NS | 1.53 [0.79-2.95] | NS |
| Ever: D4T | 1.6 [0.72-3.57] | NS | 1.82 [0.49-6.78] | NS | 2.09 [0.72-6.05] | NS |
| Ever: DDC | 0 [0-Inf] | NS | 0 [0-Inf] | NS | 0 [0-Inf] | NS |
| Ever: DDI | 1.32 [0.45-3.93] | NS | 2.76 [0.72-10.48] | NS | 0.64 [0.08-5.14] | NS |
| Ever: IDV | 1.72 [0.69-4.31] | NS | 1.32 [0.28-6.19] | NS | 2.77 [0.81-9.42] | NS |
| Ever: RTV | 1.36 [0.8-2.3] | NS | 1.33 [0.49-3.64] | NS | 1.6 [0.85-3.02] | NS |
| Ever: ZDV | 1.12 [0.62-2] | NS | 0.99 [0.35-2.84] | NS | 1.36 [0.67-2.76] | NS |
| Ever: RAL | 1.14 [0.46-2.79] | NS | 0.53 [0.07-4.09] | NS | 1.81 [0.63-5.23] | NS |
| Ever: BIC | 1.27 [0.48-3.37] | NS | 0 [0-Inf] | NS | 1.99 [0.69-5.68] | NS |
| Ever: DTG | 1.44 [0.89-2.32] | NS | 1.31 [0.58-2.97] | NS | 1.48 [0.82-2.67] | NS |
| Ever: EVG | 0.77 [0.38-1.55] | NS | 0.79 [0.23-2.73] | NS | 0.75 [0.32-1.76] | NS |
| Ever: EFV | 0.93 [0.54-1.61] | NS | 1.14 [0.47-2.77] | NS | 0.8 [0.4-1.6] | NS |
| Ever: TAF | 1.03 [0.64-1.67] | NS | 0.49 [0.18-1.34] | NS | 1.39 [0.78-2.47] | NS |
| Ever: TDF | 0.83 [0.49-1.42] | NS | 0.46 [0.19-1.09] | NS | 1.13 [0.57-2.25] | NS |

**Supplementary Table 5.** Associations between fibrosis and demographics, comorbidities, laboratory measurements, HIV-specific characteristics, and ART-exposure for the total population (columns “All”), lean participants (columns “Lean”) and overweight and obese participants (columns “Overweight and obese”). Effect estimates are presented as odds ratios with 95% confidence intervals. The odds ratios for demographics are not corrected for any confounders. Age and fat layer thickness were added to the logistic regression models with comorbidities, HIV-specific characteristics, and ART exposure. For the models with laboratory assessments, we also added lipid lowering therapy as a confounders. Abbreviations: CMV = cytomegalovirus, T2DM = type 2 diabetes mellitus, HAV = hepatitis A virus, HBV = hepatitis B virus, HCV = hepatitis C virus, HDL = high density lipoprotein, LDL = low density lipoprotein, VLDL = very low density lipoprotein, ALT = alanine transaminase, AST = aspartate aminotransferase, ALP = alkaline phosphatase, GGT = gamma-glutamyl transferase, LDH = lactate dehydrogenase, WOT = way of transmission, MSM = men who have sex with men, ART = antiretroviral therapy, NRTI = nucleoside reverse transcriptase inhibitor, NtRTI = nucleotide reverse transcriptase inhibitor, NNRTI = non-nucleoside reverse transcriptase inhibitor, PI = protease inhibitor, INSTI = integrase strand transfer inhibitor, d-drugs = dideoxynucleoside analogs, D4T = stavudine, DDC = zalcitabine, DDI = didanosine, IDV = indinavir, RTV = ritonavir, ZDV = zidovudine, RAL = raltegravir, BIC = bictegravir, DTG = dolutegravir, EVG = elvitegravir, EFV = efavirenz, TAF = tenofovir alafenamide, TDF = tenofovir disoproxil fumarate.

**Supplementary Figure 5**. Results of multivariable analysis for the associations between steatosis and demographics, comorbidities, laboratory measurements, HIV-specific characteristics, and ART-exposure for the total population (purple), lean participants (green) and overweight and obese participants (orange). All variables that showed significant associations, including the demographic variables BMI, fat layer thickness and age, were included in the final multivariate logistic regression analysis. For steatosis in the lean population, the variable “ever exposed to D-drugs” was removed because of the multicollinearity with the variable “ever exposed to D4T”.. Effect estimates are presented as odds ratios with 95% confidence intervals. Variables with infinite confidence intervals are removed from the figure. Closed circles denote significant P-values (< 0.05). Abbreviations: HBV = hepatitis B virus, VLDL = very low density lipoprotein, RAL = raltegravir, ALT = alanine transaminase, HCV = hepatitis C virus, T2DM = type 2 diabetes mellitus, ZDV = zidovudine, IDV = indinavir, D4T = stavudine, INSTI = integrase strand transfer inhibitors, ART = antiretroviral therapy, WOT = way of transmission, MSM = men who have sex with men.

|  |  | | BMI classification | | | |
| --- | --- | --- | --- | --- | --- | --- |
|  | **Total population** | | **Lean PLHIV** | | **Overweight and obese PLHIV** | |
|  | **OR** | **P-value** | **OR** | **P-value** | **OR** | **P-value** |
| ALT | 1.26 [0.9-1.75] | NA | 2.85 [0.86-9.48] | NA | 1.24 [0.86-1.77] | NA |
| Age | 1.93 [1.19-3.13] | NA | 0.89 [0.25-3.17] | NA | 0.97 [0.49-1.92] | NA |
| BMI | 1.29 [1.1-1.52] | NA | 1.72 [0.86-3.45] | NA | 1.21 [0.97-1.51] | NA |
| Black ethnicity | 0.26 [0.05-1.29] | NA | NA | NA | 1.1 [0.11-10.97] | NA |
| CD4 at enrollment | 1.18 [1.01-1.38] | NA | 1.83 [1.16-2.89] | NA | NA | NA |
| CD8 at enrollment | 1 [0.89-1.12] | NA | 0.73 [0.51-1.06] | NA | NA | NA |
| Ever: RAL | 3.17 [0.42-23.97] | NA | 187.17 [1.5-23432.25] | NA | NA | NA |
| Fatlayer thickness | 2.92 [0.93-9.14] | NA | 15.92 [0.43-596.22] | NA | 1.88 [0.32-10.92] | NA |
| Glucose | 1.48 [0.72-3.03] | NA | NA | NA | 1.25 [0.53-2.9] | NA |
| HDL | 1.41 [0.26-7.66] | NA | NA | NA | NA | NA |
| LDL | 0.69 [0.24-1.98] | NA | NA | NA | NA | NA |
| Past HBV infection | 0.3 [0.1-0.87] | NA | NA | NA | NA | NA |
| T2DM | 0.7 [0.01-76.37] | NA | 5.41 [0.17-172.18] | NA | NA | NA |
| Triglycerides | 3.85 [1.56-9.54] | NA | 5.02 [0.75-33.7] | NA | NA | NA |
| ART duration | NA | NA | 0.99 [0.71-1.39] | NA | NA | NA |
| Current: Duotherapy | NA | NA | 14.37 [0.24-868.46] | NA | NA | NA |
| Ever: D4T | NA | NA | 0.04 [0-9.59] | NA | NA | NA |
| Ever: IDV | NA | NA | 10.03 [0.05-1925.11] | NA | 13013072.02 [0-Inf] | NA |
| Ever: INSTI | NA | NA | 0.67 [0.05-8.83] | NA | NA | NA |
| Lipid lowering therapy | NA | NA | 27.32 [0.59-1255.99] | NA | NA | NA |
| Myocardial infarction | NA | NA | 27.5 [0.12-6356.9] | NA | NA | NA |
| Past HBV Infection | NA | NA | 0 [0-Inf] | NA | NA | NA |
| Cumulative: EVG | NA | NA | NA | NA | 1.55 [0.99-2.44] | NA |
| Female sex | NA | NA | NA | NA | 0.27 [0.04-1.91] | NA |
| VLDL | NA | NA | NA | NA | 2.27 [0.18-28.04] | NA |

**Supplementary Table 6.** Results of multivariable analysis for the associations between steatosis and demographics, comorbidities, laboratory measurements, HIV-specific characteristics, and ART-exposure for the total population (left two columns), lean participants (middle two columns)) and overweight and obese participants (right two columns). All variables that showed significant associations, including the demographic variables BMI, fat layer thickness and age, were included in the final multivariate logistic regression analysis. For steatosis in the total population, the cumulative duration of D4T was removed from the model because of the large number of missing values. Effect estimates are presented as odds ratios with 95% confidence intervals. Abbreviations: ALT = alanine transaminase, RAL = raltegravir, HBV = hepatitis B virus, HCV = hepatitis C virus, T2DM = type 2 diabetes mellitus, VLDL = very low density lipoprotein, ART = antiretroviral therapy, D4T = stavudine, IDV = indinavir, INSTI = integrase strand transfer inhibitors.

**Supplementary Figure 6**. Results of multivariable analysis for the associations between fibrosis and demographics, comorbidities, laboratory measurements, HIV-specific characteristics, and ART-exposure for the total population (purple), lean participants (green) and overweight and obese participants (orange). All variables that showed significant associations, including the demographic variables BMI, fat layer thickness and age, were included in the final multivariate logistic regression analysis. Effect estimates are presented as odds ratios with 95% confidence intervals. Variables with infinite confidence intervals are removed from the figure. Closed circles denote significant P-values (< 0.05). Abbreviations: ALT = alanine transaminase, HDL = high density lipoprotein, T2DM = type 2 diabetes mellitus, VLDL = very low density lipoprotein, LDH = lactate dehydrogenase.

|  |  | | BMI classification | | | |
| --- | --- | --- | --- | --- | --- | --- |
|  | **Total population** | | **Lean PLHIV** | | **Overweight or obese PLHIV** | |
|  | **OR** | **P-value** | **OR** | **P-value** | **OR** | **P-value** |
| ALT | 1.17 [1.05-1.3] | 0.003 | 1.24 [1.05-1.46] | 0.013 | 1.43 [1.12-1.82] | 0.004 |
| BMI | 1.1 [1.02-1.2] | 0.017 | NA | NA | 1.13 [0.94-1.36] | NS |
| Fatlayer Thickness | 1.87 [0.86-4.07] | NS | NA | NA | 2.21 [0.36-13.39] | NS |
| Glucose | 1.09 [0.88-1.35] | NS | NA | NA | 1.21 [0.87-1.68] | NS |
| HDL | 0.15 [0.04-0.56] | 0.005 | NA | NA | 0.02 [0-0.48] | 0.017 |
| LDL | 0.67 [0.34-1.3] | NS | 0.22 [0.06-0.81] | 0.023 | NA | NA |
| Myocardial infarction | 1.52 [0.35-6.65] | NS | NA | NA | 34557866.13 [0-Inf] | NS |
| T2DM | 1.74 [0.55-5.5] | NS | NA | NA | 2.43 [0.41-14.41] | NS |
| VL Zenith | 1 [1-1] | 0.010 | 1 [1-1] | NS | 1 [1-1] | NS |
| VLDL | NA | NA | 2.31 [0.25-21.55] | NS | NA | NA |
| Cholesterol | NA | NA | NA | NA | 0.9 [0.46-1.79] | NS |
| LDH | NA | NA | NA | NA | 0.76 [0.61-0.95] | 0.018 |

**Supplementary Table 7**. Results of multivariable analysis for the associations between fibrosis and demographics, comorbidities, laboratory measurements, HIV-specific characteristics, and ART-exposure for the total population (left two columns), lean participants (middle two columns)) and overweight and obese participants (right two columns). All variables that showed significant associations, including the demographic variables BMI, fat layer thickness and age, were included in the final multivariate logistic regression analysis. Effect estimates are presented as odds ratios with 95% confidence intervals. Abbreviations: ALT = alanine transaminase, HDL = high density lipoprotein, T2DM = type 2 diabetes mellitus, VLDL = very low density lipoprotein, LDH = lactate dehydrogenase.

**
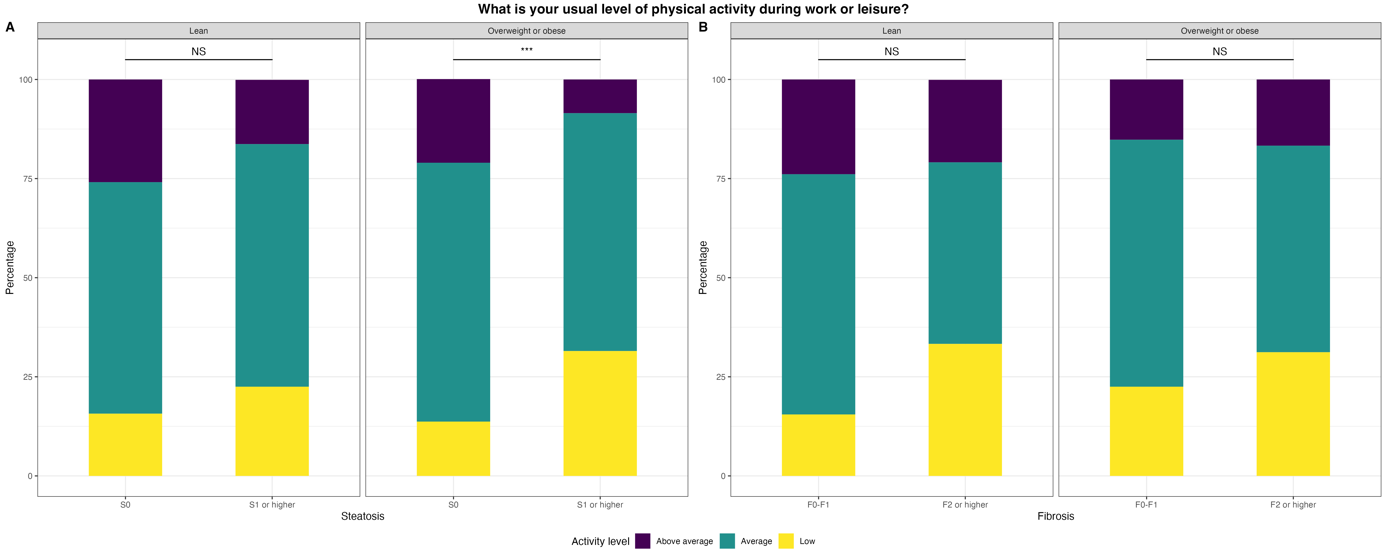
**

**Supplementary Figure 7**. Barplots showing the level of physical activity for participants (A) with and without steatosis and (B) with and without fibrosis, categorized by subgroup. Participants indicated the level of physical activity according to the question “What is your usual level of physical activity during work or leisure?”. The legend at the bottom indicates the level of physical activity according to the participants. On top of the barplots, a significance level is shown for a comparison by chi-square test for the level of physical activity between (A) participants with and without steatosis, and (B) participants with and without fibrosis: *: P-value 0.01 – 0.05, **: P-value 0.001 – 0.05, and ***: P-value < 0.001.


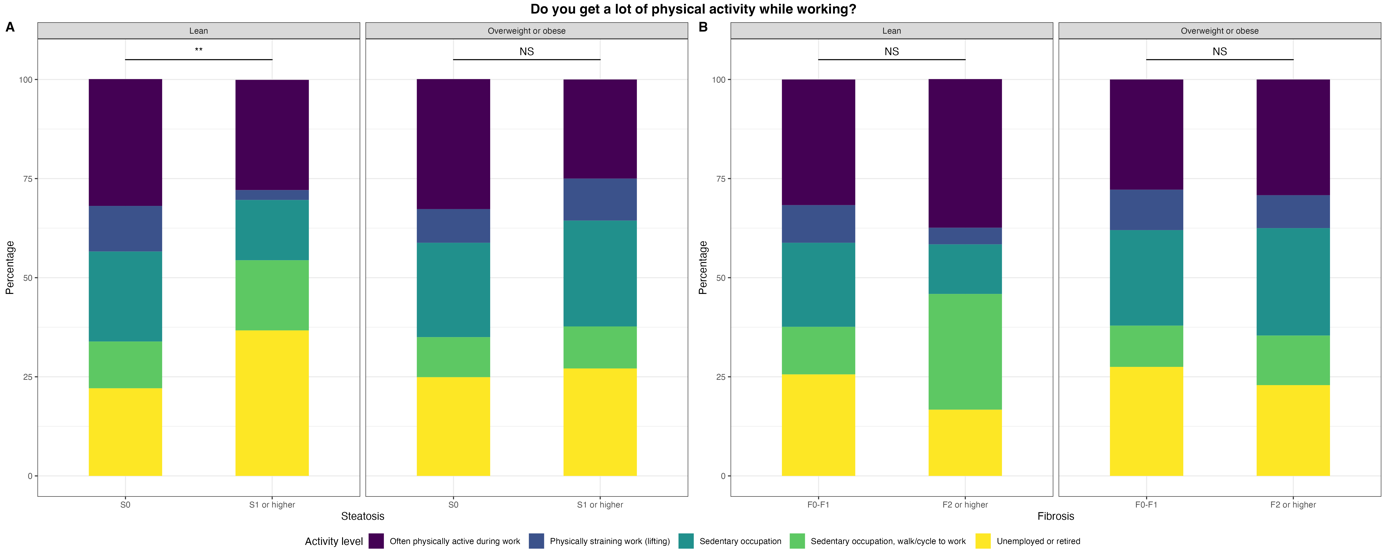


**Supplementary Figure 8**. Barplots showing the level of physical activity while working for participants (A) with and without steatosis and (B)with and without fibrosis, categorized by subgroup. Participants indicated the level of physical activity according to the question “Do you get a lot of physical activity while working?”. The legend at the bottom indicates the level of physical activity according to the participants. On top of the barplots, a significance level is shown for a comparison by chi-square test for the level of physical activity between (A) participants with and without steatosis, and (B) participants with and without fibrosis: *: P-value 0.01 – 0.05, **: P-value 0.001 – 0.05, and ***: P-value < 0.001.

**References**

1. Soininen P, Kangas AJ, Wurtz P, Suna T, Ala-Korpela M. Quantitative serum nuclear magnetic resonance metabolomics in cardiovascular epidemiology and genetics. Circ Cardiovasc Genet. 2015;8(1):192-206.

2. Verna EC. Non-alcoholic fatty liver disease and non-alcoholic steatohepatitis in patients with HIV. Lancet Gastroenterol Hepatol. 2017;2(3):211-23.

3. Bischoff J, Gu W, Schwarze-Zander C, Boesecke C, Wasmuth JC, van Bremen K, et al. Stratifying the risk of NAFLD in patients with HIV under combination antiretroviral therapy (cART). EClinicalMedicine. 2021;40:101116.
